# An Insight into the Triabin Protein Family of American Hematophagous Reduviids: Functional, Structural and Phylogenetic Analysis

**DOI:** 10.3390/toxins8020044

**Published:** 2016-02-15

**Authors:** María J. Hernández-Vargas, Carlos E. Santibáñez-López, Gerardo Corzo

**Affiliations:** Departamento de Medicina Molecular y Bioprocesos, Instituto de Biotecnología, Universidad Nacional Autónoma de México (UNAM), Apartado Postal 510-3, Cuernavaca, Morelos 61500, Mexico; majohv@gmail.com (M.J.H.-V.); cae@ibt.unam.mx (C.E.S.-L.)

**Keywords:** triabin, antihemostatic proteins, coagulation, platelet aggregation, saliva, Triatominaes

## Abstract

A transcriptomic analysis of the saliva of *T. pallidipennis* together with a short proteomic analysis were carried out to reveal novel primary structures of the lipocalin/triabin protein families in this reduviid. Although triabins share some structural characteristics to lipocalins and they are classified as in the calcyn/lipocalin superfamily, triabins differ from lipocalins in the direction of β-strands in the general conformation of the β-barrel. The triabin protein family encompasses a wide variety of proteins, which disrupt the hemostasis of warm-blooded animals. Likewise, the function of proteins classified as triabins includes proteins that are carriers of small molecules, protease inhibitors, binders of specific cell-surface receptors as well as proteins that form complexes with other macromolecules. For example, triabin and pallidipin from the saliva of *T. pallidipennis* are thrombin and platelet aggregation inhibitors, respectively; triplatin from *T. infestans* binds to thromboxane A2; and nitrophorin from *Rhodnius prolixus* carries nitric oxide. Therefore, based on 42 new transcriptome sequences of triabins from the salivary glands of *T. pallidipennis* reported at present, and on triabin sequences of other American hematophagous reduviids already reported in the literature, subfamilies of triabins were proposed following phylogenetic analyses and functional characterization of triabin members. Eight subfamilies of proteins were recognized with known functions, which were the nitrophorin and amine binding proteins, *Rhodnius prolixus* aggregation inhibitor, triafestin, triatin, dipetalodipin and pallidipin, triplatin and infestilin, dimiconin and triabin, and procalin subfamilies. Interestingly, 70% of the analyzed sequences came from these eight subfamilies because there was no biological function associated with them, implying the existence of a vast number of proteins with potential novel biological activities.

## 1. Introduction

The Triatominae subfamily consists of hematophagous insects that are represented by 140 species [1,2,3]. The species of this family are usually vectors of Chagas disease, a human disease caused by *Trypanosoma cruzi*, which is a protozoan that lives in the digestive system of the insect. *T. cruzi* is deposited on the human skin through the insect’s feces during blood-feeding. The protozoan can then penetrate through skin lesions causing Chagas disease, which affects the population of tropical and subtropical countries. It is estimated that 16–18 million people are infected by this protozoan, which is responsible of 50,000 deaths annually [4]. Hematophagous reduviids have evolved along the American continent, where they have attracted significant attention because, as mentioned above, they are vectors of Chagas disease, and also because their saliva contains protein components that interrupt the hemostasis of warm-blooded animals; so potentially, they are of great therapeutic and pharmaceutical interest. The saliva of Triatominae disrupts principally the physiological mechanisms of vasoconstriction, aggregation and coagulation.

In brief, vasoconstriction is the first response to a vascular injury, and is activated by second messengers like epinephrine that interact with adrenergic receptors and trigger the release of Ca^2+^ of the sarcoplasmic reticulum that activates the calmodulin system in order to induce myogenic contractions. There are other second messengers that can activate the vasoconstriction in blood vessels like tromboxane A_2_ (TA_2_) that interacts with prostanoid receptors and shows effects also in hypertension [5,6].

Second, the activation and aggregation of blood platelets are mediated by several steps where the von Willebrand factor (vWF) leads to the adhesion of the platelets [7], and in turn with integrins which are activated [8,9] to interact with collagen, elastin, laminin, vitronectin, vWF, fibrinogen and fibronectin to improve platelet adhesion. The ligands that can activate this integrin are ADP, thromboxane A_2_ (TA_2_) and thrombin [10,11]. There are also other intrinsic ligands that lead to platelet shape changes and secretion of pro-coagulant molecules [12].

**Figure 1:**
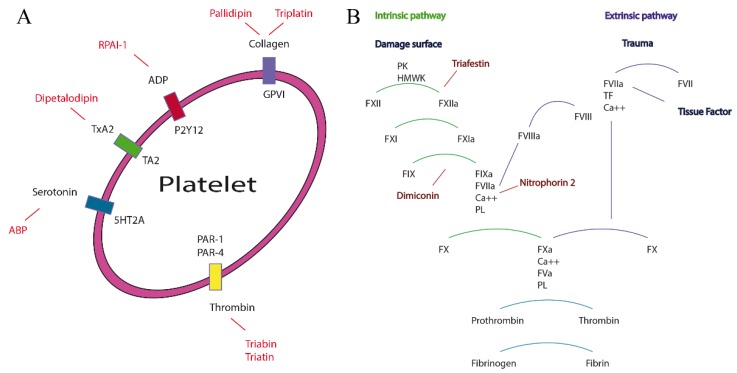
Proteins from the salivary glands of triatomines implicated in platelet aggregation and inhibition of blood coagulation. (**A**) Proteins involved in platelet aggregation; (**B**) Proteins involved in the inhibition of the blood coagulation pathway.

Finally, the blood coagulation consists in a serine proteases cascade that use cofactors for activation, and it is led by two pathways; (1) the intrinsic one that is composed of prekallikrein and is activated by subendotelial structures, collagen and basal membrane [13]; and (2) the extrinsic pathway, which is composed of factor VII (FVII) and is activated by tissue factor (TF). This pathway is the first response to coagulation and the fastest one (Figure 1). These two pathways lead to the activation of thrombin and, consequently, to the cleavage of fibrinogen in to fibrin in order to make the stacking of the thrombus [14].

Proteins of the triabin family from Triatominae inhibit vasoconstriction, aggregation and coagulation (Figure 1). The saliva of triatomine species also represents a great source of possible novel functional proteins that could be of pharmacological interest, with possible better results than currently commercially available, like in the case of NP2 that shows the same effect as heparin but in lower concentrations.

The triabins were first classified in the lipocalin family because of their strong 3D structural similarities that consist in eight-stranded antiparallel β-barrels [1]; however, later it was considered as a distinctive family strongly related to lipocalins, and considered a member of the superfamily of calycins [2]. Also, Pfam considers both lipocalins and triabin families distinctive [15]. Triabins, similar to lipocalins, act as enzyme inhibitors and form complexes with other proteins makers, and carrier proteins are represented by nitrophorins, which have global similarities to triabins despite that Pfam has separated them [2].

In this contribution, 42 novel triabin primary structures obtained from the saliva of *T. pallidipennis* are reported. Also, based on the new triabin primary structures reported at present, and on triabin and nitrophorins sequences of other American hematophagous reduviids already described in the literature, subfamilies of triabins are proposed following a phylogenetic analyses and functional characteristics of triabin members, which until now represent only proteins that act as enzyme inhibitors and those that make complexes. Therefore, the subfamilies of triabins here suggested are based on structural and functional similarities, including the nitrophorins, which share structural characteristics with amine binding proteins and triabins [16,17], here regarded as a subfamily of triabins.

## 2. Results and Discussion

### 2.1. Proteomic and Transcriptomic Analysis of Triabins in Triatoma pallidipennis

**Figure 2:**
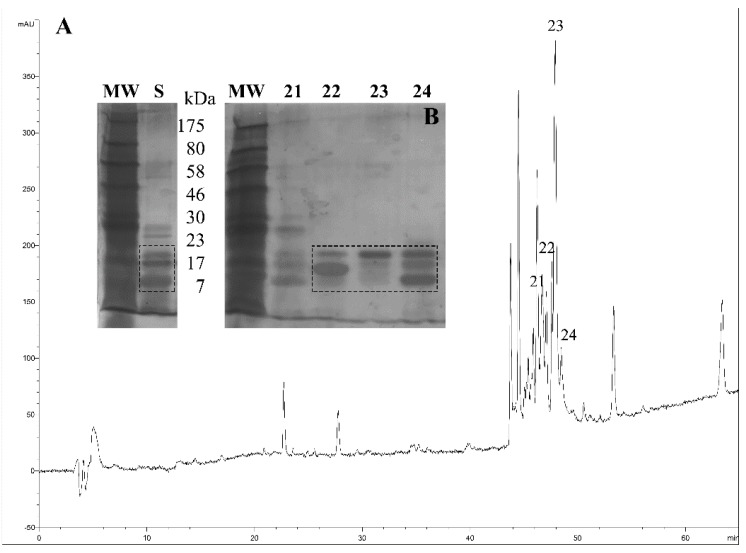
Reverse-phase chromatographic profile of the saliva from *T. pallidipennis*. (**A**) The saliva (230 μg) was fractionated using a linear gradient that employs aqueous solutions of 0.1% TFA from 0 to 60% acetonitrile; (**B**) Inset, some HPLC fractions were further separated using SDS-PAGE and revealed under silver stain. The inset labels; MW represents the molecular weight of protein markers in kDa; the letter S represents the protein components of the saliva from *T. pallidipennis*; and, the numbers 21, 22, 23 and 24 denote the collected fractions from reverse phase HPLC. Fractions 22, 23 and 24 were additionally analyzed using MS/MS.

*Triatoma pallidipennis* from the State of Morelos, Mexico were collected and maintained under controlled conditions of moisture and temperature. The saliva of several individuals was obtained by manual stimulation. The saliva was vacuum dried before chromatographic separation and stored a low temperature. The saliva was separated using a reverse phase HPLC system, and the chromatographic fractions were collected (Figure 2A). All fractions were further separated using SDS-PAGE, and special attention was given to the collected protein fractions 22, 23 and 24, which were the most highly expressed saliva proteins (Figure 2B, discontinued squares). Also, the molecular weight of proteins in fractions 22–24 motivated the decision to select them for proteomic analysis. Therefore, fractions 22–24 were trypsinized and then analyzed using MS/MS.

Altogether, the transcriptome of the salivary glands from *T. pallidipennis* was obtained by massive pyrosequencing using the Illumina mRNA-Seq technology and *de novo* assembly. A total of 65,472 transcript-derived contigs were assembled, from which 28,000 transcripts presented a clear open reading frame, and 25,681 sequences were annotated according to Swiss prot and Pfam databases.

The tryptic peptide sequences of fractions 22, 23 and 24, obtained by MS/MS, were used to find their complete primary structures based on the transcriptome of *T. pallidipennis* (Table 1).

**Table 1:** Protein sequences obtained based on MS/MS analysis of fractions 22, 23 and 24 from the saliva of *T. pallidipennis*.

ID/Fraction	Amino Acid Sequences ^a^	MS/MS ^b^	Score ^c^	Exp. ^d^	aa ^e^	MW (kDa) ^f^
27479c2s7 Fraction 22	MKVIIAATLLGILMHAFAKECELMPPASNFDSEKYFDIPHVYVTHSRNGPKEQVCREYNTTKIQGDTTPYTVVTSDYKIRGETHHSQLKCTNTPKNGGKGQFSVECEISNGNGGNKKKVQFETSVFATDYKNYALLQSCTKTESGIADDVLLLQTKKEGVDPGVTSVLK$\underline{\mathrm{SVNWSLDDWFSR}}$SKVNCDNMK *	1	67	0.018	179	20.3
24833c0s1 Fraction 22, 23 and 24	MKTITAVTFFGILTYAFADTIKYGGQTCQQQVPIMKDFDVER$\underline{\mathrm{FFSGSWSLTHSTR}}$SPRVTESTICRDYELKVHENGTFGVTYGYFENSGRNNRYDINCLGTRSDQPGLSYFDCYLTNARGEKTHTRIDGYFVTTDYDNYCLVYRCVTSDDKFEDNVFVLYRNKNYIPKDEEVKKIIEPYGLGLEQFISRKDATCTNK *	1	92	5.40 × 10^−5^	179	20.8
24833c0s2 Fraction 22	MKTIIAVTFFGILTSAFIVTAEKLEYGKGVCQNNKLDGLVNLNAQKFFSGTWYLTHATKSTRVTLSTICRDFEPKQKEDGTFEVTYGYYENGGKQNHYDVSCSGTQDKTRLDIFNFDCKSNNERGETTSFHIDGSFLATDYDSYGVVYR$\underline{\mathrm{CVTTGTLTEDNVFLIHR}}$QKNPSDEEVTKILTHYGLSLGDVISRKDATCTNK *	1	42	6.5	179	20.3

^a^ In italics are represented the signal peptide; underlined are the mature protein; and in bold red the trypsinized peptide fragments found using MS/MS; * end of protein by stop codon. ^b^ Number of tryptic peptides found using MS/MS. ^c^ Score means the probability of either the peptide masses or the MS/MS fragment ion masses is a random event, the higher the number the less random the event is. ^d^ Expectancy means the number of protein matches with equal or better scores that are expected to occur by chance alone, the smaller the number, the less random the event is (http://www.matrixscience.com/help/scoring_help.html). ^e^ Number of amino acids in the protein. ^f^ Theoretical molecular mass of the mature protein.

According to the values of score and expectancy, the less random event was for the protein 24833c0s1, and the most random event was for protein 24833c0s2. Besides, the fact that the MS/MS sequenced tryptic peptides match a segment of the primary structure of the recovered transcripts made the proteomic correlation accurate. Even though the proteomic analysis of fractions 22–24 revealed only three proteins sequences that were correlated to the transcriptome, it exposed other primary structures of the triabin family. Table S1 shows the 42 primary structures of expected mature proteins from the salivary glands of *T. pallidipennis*. In order to include these new primary structures with those reported from the saliva of other triatomines, a compilation and categorization of the triabin family of such proteins based on functional, structural and phylogenetic analysis was performed and is presented hereafter.

### 2.2. Functional and Structural Features in Lipocalins and Triabins

**Figure 3:**
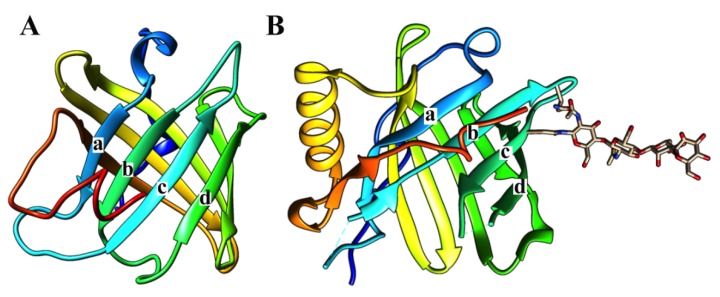
Three-dimensional structures of triabin and lipocalin. Triabin (**left**, PDB:1AVG) is a thrombin inhibitor where four of its β-strands has the directions down (**a**), down (**b**), up (**c**), up (**d**) forming half of the β-barrel. On the other hand, the β-strands of lipocalin (**right**, PDB:3S26) has the directions down (**a**), up (**b**), down (**c**), up (**d**) forming half of the β-barrel. The lipocalin structure here is coupled to an *N*-linked glycan.

According to Flower and collaborators [2,18], the lipocalin family contain a protein barrel of eight well conserved hydrogen-bonded antiparallel β-strands that has an internal cavity and an external loop scaffold, which has the capacity to bind ligands of different sizes, shapes and chemical characteristics. Their structural characteristics explain the diverse biological activities of lipocalins as anticoagulants, antiplatelets and vasodilators (Figure 3).

**Figure 4:**
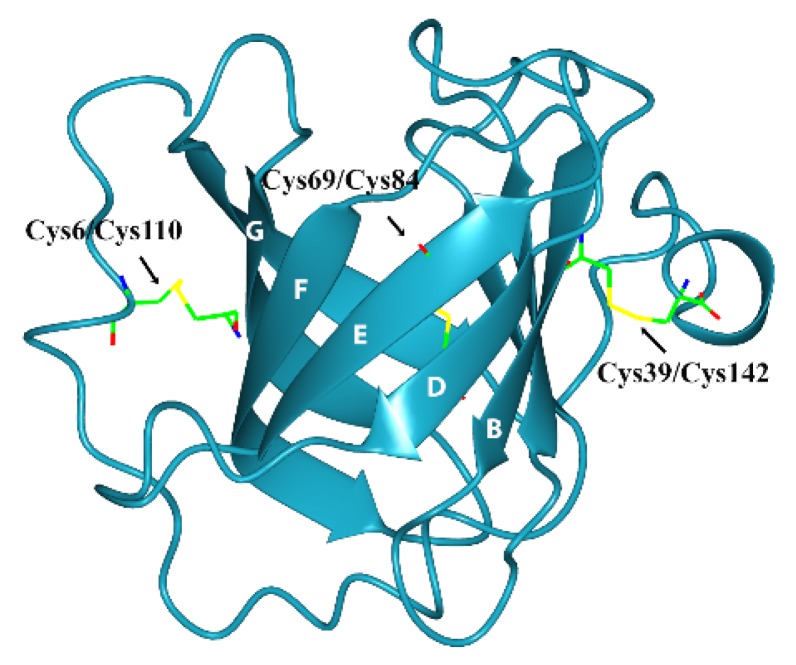
Three-dimensional structure of triabin (PDB:1AVG) including the disulfide bonds.

All of the above biological and structural features are shared with most of the proteins of the triabin family with some structural exceptions; that is, the triabin family shows an exchange in the B and C β-strands (Figure 3A) that breaks the antiparallel pattern [1] of the position of the β-sheet going down, down, up, up, down, up, down and up instead of up and down the lipocalin β-strands (Figure 3B). The stability of the β-barrel in triabin depends of the disulfide bonds that connect the strands D and E by Cys69/Cys84, the *N*-terminal segment with the strand G by Cys6/Cys110, and the C-terminal helix with the strand B by Cys39/Cys142 (Figure 4) [1].

**Figure 5:**
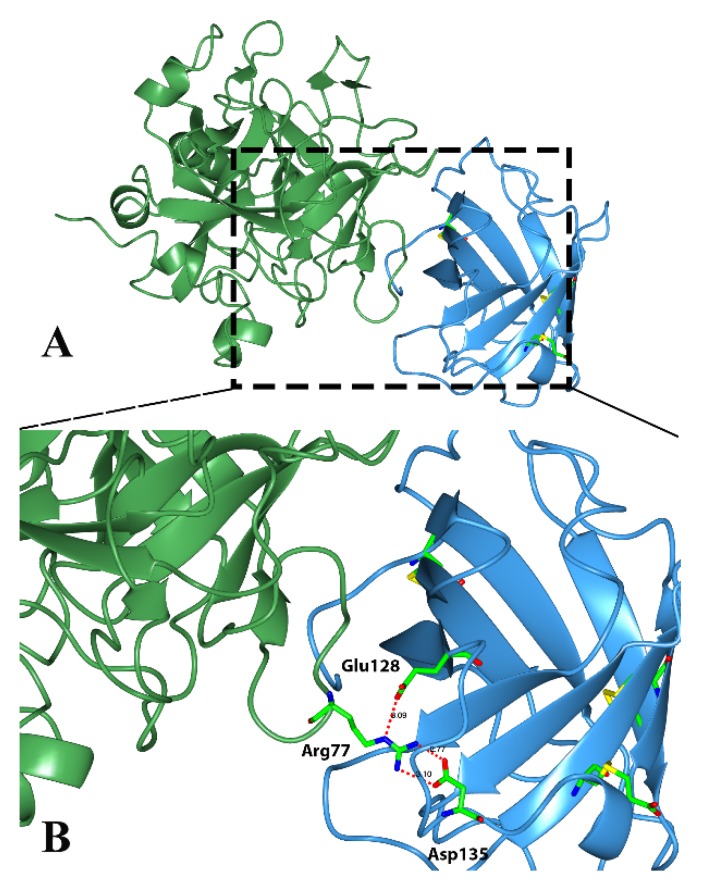
Three-dimensional structure of the thrombin (green)—triabin (blue) complex showing the hydrogen bonds formed. (**A**) Triabin—thrombin complex; (**B**) A close up of hydrogen bonds between Asp135 and Glu128 from triabin and Arg77 from thrombin.

Classical triabins are 142 residues long and interact with thrombin to the fibrinogen recognition exosite, which comprises around 19 residues from triabin and 13 from thrombin. There are three main surfaces that interact; (1) the hydrophobic interaction between the residues Ile99, Leu108, Val126 and Phe109 from triabin with Leu65 and Met84 from thrombin; (2) the polar interaction of Glu9 from triabin with Lys36 and Lys109 from thrombin; and (3) hydrogen bonds between Asp135 and Glu128 from triabin and Arg77 from thrombin as shown in Figure 5 [1].

### 2.3. Phylogenetic Analysis of Triabins Including Nitrophorins

**Figure 6:**
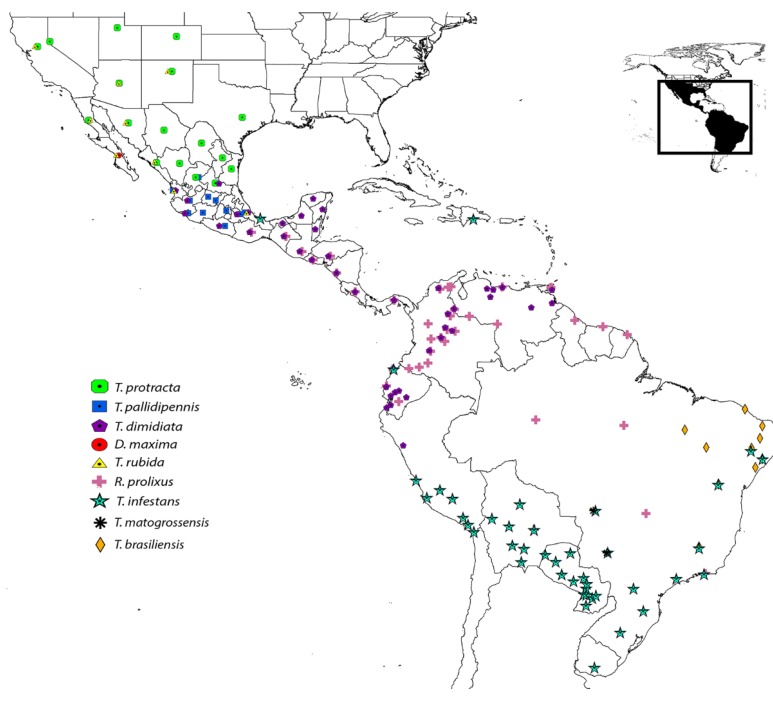
Distribution of the nine triatomine species in America used for the phylogenetic analysis (modified according to [20]).

A phylogenetic analysis was performed to determine if triabins could be subsequently divided into subfamilies by functional or phylogenetic affinity and if nitrophorins (NP) belong to the family of triabins [17]. Proteins were retrieved using firms of amino acid sequences (family assignments) within the Pfam database from the triabin and nitrophorin families, and also from the primary structures found in the transcriptome analysis of the saliva of *T. pallidipennis*. The phylogenetic analysis was based on 339 amino acid sequences (from 63 to 256 amino acids long, Table S2) from the saliva of nine triatomine species distributed from South to North America (Figure 6). The phylogenetic analysis as well included six protein sequences from *Blattella germanica* (German cockroach, A9XFW8, B7TYB1, B7TYB2, C3RWZ4, C3RWZ5 and P54962) and one protein from *Naegleria gruberi* (Amoeba, D2UZV1), all enclosed in the triabin family. Originally, the six proteins from the German cockroach were considered as lipocalins [19]; however, Pfam consider them as triabins [15]. Therefore, our analysis would explore the phylogenetic affinity of these sequences, if they would be regarded as lipocalins, or differently. Nevertheless, our conclusions should not be considered definitive, but exploratory (since not all calycins are included), and may serve as a basis for future studies. The Pfam database was selected to test the phylogenetic positions within this large family. In addition, three protein sequences of human lipocalins were used to orientate the phylogenetic tree. We considered these human lipocalins because they are better characterized than other lipocalins (e.g., those from insects).

The protein sequence matrix was aligned using MAFFT (Multiple Alignment using Fast Fourier Transform in its online version at http://mafft.cbrc.jp/alignment/software/; [21,22]) that allows a quick search for conserved motifs and provides an iterative process to refine the results. The L-INS-I strategy was selected because this method is recommended for sequences with conserved domains and long gaps regions such as in our data [21]. The best fitting protein evolution model (or amino acid replacement model) was selected using the software ProtTest 3 [23,24]. Among the 120 models currently available, and according to the Akaike information criterion, the selected model was WAG + G (the Whelan and Goldman model; an empirical model for globular protein evolution, plus a Gamma distribution of probabilities [22]). A Bayesian inference analysis was conducted using the algorithm implemented in BEAST version 1.8 (Bayesian Evolutionary Analysis Sampling Tree; [25]), a software using MCMC (Markov chain Monte Carlo) for sampling trees from probability distribution, resulting in the most likely phylogenetic tree given our data and our chosen model, and with the highest branch posterior probabilities. The analysis consisted of 40 million generations, sampling every 1000 generations and discarding those before stationarity (4 million generations) using the burn-in option in TreeAnotator (included in the BEAST software package). Our resulted topology was then edited in FigTree 1.3.1 (http://tree.bio.ed.ac.uk/software/figtree/). The tree was rooted using the sequences of human lipocalins (gi|7245434, gi|308387777 and gi|313856) because they have similar 3D protein structures.

**Figure 7:**
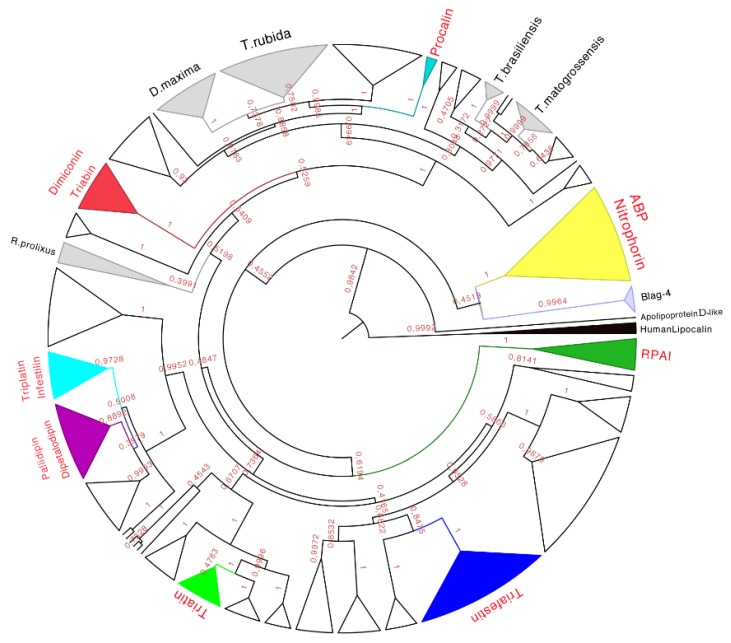
Phylogenetic tree obtained from the Bayesian analysis of 339 amino acid sequences of triabins and nitrophorins, using human lipocalins (in black) to root the tree. In yellow is the nitrophorin clade, in dark green is the *Rhodnius prolixus* aggregation inhibitor-1 (RPAI) clade, in blue is the triafestin clade, in light green is the triatin clade, in purple is the pallidipin and dipetalodipin clade, in cyan is the triplatin clade, in red is the triabin and dimiconin clade, in aqua blue is the procalin clade, and in white are the proteins that may form clades but do not have strong identities with the previously characterized proteins. Finally, in gray are the protein clades that have no biological function reported in respect to previously characterized ones, and that have amino acid sequences unique to those species. The names in red represent triabin proteins that have been functionally characterized.

The phylogenetic analysis of the 339 amino acid sequences recovered the monophyly (with high posterior probabilities over 98%) of the family of triabins, including the nitrophorins and the triabins from *Blatella germanica* (as suggested by Pfam, [15]). The triabin from *Naegleria gruberi* was recovered as a sister group to the rest of the triabins (Figure 7), suggesting that triabins have evolved independently in different organisms (Reduviidae *vs.* Blattidae).

Taking into account the posterior probability of each clade, sets of amino acid sequences were selected to determine the motifs obtained by the Multiple Em for Motif Elicitation (MEME 4.10.0) and by the Motif Alignment & Search Tool (MAST), searching for sets of 10 representative motifs in each sequence belonging to the clade (MEME) with at least once appearance per sequence. MAST was used to determine if the selected motif was represented only in the clade in order to keep a unique signature for each clade.

Using these bioinformatics tools, it was able to establish subfamilies of proteins structurally similar between them that biologically disrupt the hemostasis of warm-blooded animals in any of their three main mechanisms such as vasoconstriction, aggregation or coagulation.

Therefore, eight subfamilies of proteins were recognized with known functions (see below), which were the (1) Nitrophorin-ABP; (2) RPAI-1; (3) Triafestin; (4) Triatin; (5) Dipetalodipin and pallidipin; (6) Triplatin and infestilin; (7) Dimiconin and triabin; and (8) Procalin subfamilies. This phylogenetic analysis also recovered 25 clades with unknown biological functions, and some of them were assigned to specific species (e.g., *D. maxima* triabins, *T. rubida* triabins), whereas others were different proteins with unknown biological functions from different species clustered together. The relationships between these newly created subfamilies were not well detailed in our phylogenetic analysis due to low posterior probabilities support values at more exclusive clades. However, the subfamilies of triafestins, triatins, triplatins-infestilins, and pallidipins-dipetalodipins were found to be more closely related to each other (grouped in a clade with 73% of the posterior probabilities) than to nitrophorins, RPAIs, procalins, and triabins and dimiconins, suggesting a phylogenetic affinity among them. The subfamily pallidipin-dipetalodipin is more related to the subfamily of triplatin-infestilin, whereas the triafestin subfamily is more related to the triatin subfamily. Procalins were a recovered grouped in a well-supported clade, and finally, four specific species clades (*D. maxima*, *T. rubida*, *T. brasiliensis* and *T. matogrossensis*) were recovered and grouped (Table S3). This hypothesis of triabin evolution could serve as a basis for future studies of those proteins to reveal their functions.

A brief abstract of the subfamilies with known biological functions is described here after.

#### 2.3.1. Nitrophorin and Amine Binding Protein Subfamily

There are five NPs characterized in the saliva of *R. prolixus*. The most abundant NP in the saliva of *R. prolixus* is NP2 [26], and within the saliva, NPs represent 50% of the total proteins [27,28]. The primordial role of these proteins during the blood ingest is to release Nitric Oxide (NO) into the blood vessel, promoting the relaxation and dilatation of the vessel, and improve its permeability [27,29]. NPs change their affinity for NO in a pH-dependent manner, increasing at pH 5 and decreasing at physiological pH 7.5. NPs are also capable of catalyzing the formation of NO from NO_2_ in a large range of pHs from pH < 4 to 7.5 [30]. The *K*_eq_ of the NPs for NO is variable from 6 to 9 at pH 7.5 and 6.9 to >9 at pH 5.5 differently to the histamine that is between 5 and 8.7 at pH 7.5. These variations depend on the NP and its specificity [31]. Once the NP is in the blood, the change of pH causes a change in the structure, meaning the NP is able to release NO and kidnap the histamine in the blood in order to inhibit its vasoconstriction effect [32]. The conformational change that makes the switch of NO for histamine is due to the loops between AB and GH β-strands; besides, these structures are responsible for the stability of the binding to the ligands [17]. NP2 has another physiological function *in vivo*. It has an anticoagulation effect interfering with the intrinsic pathway in early stages [33] binding to the FIX and FIXa active site [34,35]. For example, 20–40 µg/mL of NP2 can reduce the thrombin formation by 50%–80%, and NP2 in concentrations of 400 µg/kg has a better effect than heparin; furthermore, in doses of 1 mg/kg it can even increase the time taken for the carotid artery occlusion [36]. NP7 is the fifth protein of this subfamily that interacts with membrane lipids, especially with l-α-phosphatidyl-l-serine (PS), important for the coagulation cascade [37,38], and show that, in the physiological condition, during the binding of NP7 to vesicles composed of 3:1 of PC:PS and 150 mM of NaCl the affinity for the vesicle is 65% with an IC_50_ ~1.2 µM in the presence of 1.5 µM of phospholipids. NP7 differs from NP2; it has five lysine residues along the helix that apparently are responsible for the binding to the phospholipids [37].

ABPs were found in the salivary gland of *R. prolixus* and they can kidnap serotonin and inhibit the interaction between the receptor 5-HT and, as a consequence, inhibit the contraction of the rat uterus in a stoichiometry 1:1 when added to the preparation [39]. ABPs can also interact with norepinephrine in the *Rattus* aortic ring avoiding its interaction with α-adrenergic receptors and inducing relaxation at a ratio of 2 µM ABP: 1 µM norepinephrine [39]. Since epinephrine is an agonist of the α_2_ adrenergic receptor, and serotonin is a second messenger for the platelet aggregation, they induce the changes in the form of the platelet. ABPs are shown to have a higher affinity for norepinephrine (*K*_d_ of 24 ± 6 nM); epinephrine affinity is about 10 times lower (*K*_d_ of 345 ± 67 nM), while its affinity for serotonin is intermediate (*K*_d_ of 102 ± 38 nM). It seems that the flexibility of the β-barrel allows the binding of several ligands [39].

**Figure 8:**
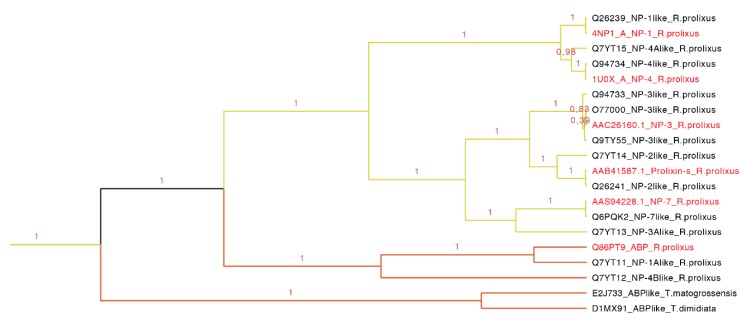
Nitrophorins and amine binding proteins expanded (yellow clade in Figure 7). NPs and AMPs are protein carriers. The clade shows the posterior probabilities, in yellow are the types of NPs and in orange the ABPs. The labels in red are the proteins that have been functionally characterized.

Our phylogenetic tree showed that nitrophorins (NPs) were separated in three groups: NP1 + NP4, NP3 + NP2 and NP7 with different affinities for distinct ligands (Figure 8). The weak posterior probability (0.45 in Figure 7, yellow clade) supporting the separation of NPs as a different family from the triabin family is not significant; therefore, our topology suggests a close phylogenetic affinity of the NPs and triabins, regarding the former as a subfamily of the latter, encompassing the carrier proteins ABP and NPs (Figure 8). Our topology (Figure 8) suggests a close phylogenetic relationship between the ABP and NPs from *R. prolixus* supported by 100% of the posterior probabilities. Both type of proteins have a conserved core in the internal cavity where the ligand nitric oxide and histamine are bound in the case of NPs and amines like epinephrine and serotonin in ABP. The ABP clade was not revealed to be monophyletic due to the exclusion of two sequences obtained from two different species (E2J733 and D1MX91 in Figure 8), which suggests that ABP and NPs are species specific.

This clade of 20 protein sequences (63–197 residues long) has two main motifs: one representing the amino sequence PRESGGTVKEALYHY with an *E*-value of 4.40 × 10^−114^, and the second representing the YTAKYRIVDKNRRE with an *E*-value of 9.70 × 10^−60^ (Figure S1 and Table S3). As observed in the tree (Figure 8), NP1 and NP4 are more related, NP3 and NP2 (prolixin-s) make a group, and the most distant NP is NP7. ABP is separated from the NPs forming a new group.

#### 2.3.2. *Rhodnius prolixus* Aggregation Inhibitor-1 Subfamily

The *Rhodnius prolixus aggregation inhibitor-1 subfamily* (RPAI-1) is a 155-residue protein that causes slow rate platelet aggregation with an IC_50_ of ~200 nM in low concentration of collagen and at 5–10 µg/mL [40]. In higher concentrations, RPAI-1 can inhibit the aggregation by TA_2_ and arachidonic acid with an IC_50_ of ~200–600 and 200–400 nM, respectively. RPAI-1 could inhibit the aggregation mediated by ADP as a nucleotide-binding molecule <1 µM [40].

**Figure 9:**
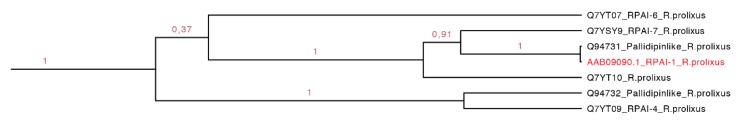
Monophyletic clade that shows seven sequences of RPAI of *R. prolixus* embedded in the same clade (green clade in Figure 7 expanded). In red, the only protein that has been functionally characterized.

As we can see in our phylogenetic tree (Figure 7, dark green clade), this group represents a monophyletic clade and it is composed of amino sequences found only in one species (supported by 100% of the posterior probabilities, Figure 9). All sequences embedded probably have similar function. The motif HPDACETFQVNGDEI was found specific for a set of seven protein sequences (134–162 residues) with an *E*-value of 2.6 × 10^−7^ (Figure S2 and Table S3).

#### 2.3.3. Triafestin Subfamily

Triafestin-1 and triafestin-2 are 188-residue proteins found in *T. infestans* salivary glands that increase the length of the intrinsic pathway of coagulation, and can affect the bradykinin generation, as well as the activity of FXIIa and the kalikrein-kinin system in a dose dependent manner [35]. They can interact with the *N*-terminal of FXII and the D5 domain of HMWK domains responsible for the biological activity in doses from 100 to 400 µM. The affinity is dependent also on the concentrations of Zn^2+^. At higher concentrations (~150 µM), triafestins have more affinity for FXII, FXIIa and HMWKa, but at lower concentrations (~25 µM) their affinities for HMWK increase. When FXII and HMWK are exposed to acidic phospholipid monolayers, their affinities decrease in a dose dependent manner, inhibiting the formation of the complex in the membrane. While the doses of the triafestins increases, the affinities of FXII and HMWK for the monolayer decreased [35].

**Figure 10:**
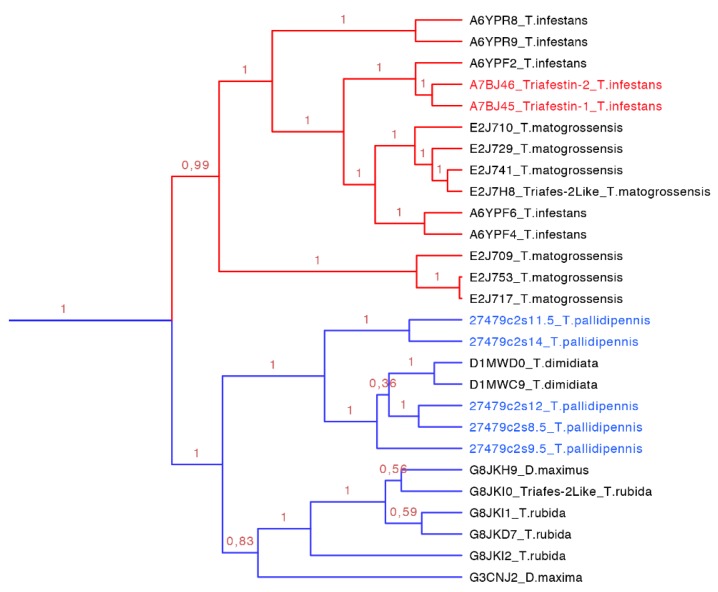
Phylogenetic tree of triafestins (blue clade expanded from Figure 7). In red is shown the clade of the amino sequences from the South American species, which proteins that have been functionally characterized, and in blue is shown the new proteins reported in this work from *T. pallidipennis* a North American species.

The triasfestins were revealed to be monophyletic with 100% posterior probabilities in our phylogenetic analysis (blue clade in Figure 7 expanded in Figure 10). This clade comprises 27 sequences from 109 to 201 amino acids separated in two well define clades (Figure 10). Interestingly, these two clades reflect the split in the distribution of the species: the first clade (red clade in Figure 10) is represented for salivary proteins from South American species, and the second clade (blue clade in Figure 10) is denoted for salivary proteins from North American species. It is noteworthy that triafestins were not grouped by species, which reflects the presence of more than one type of triafestins in the saliva of one species (e.g., the triafestins from *T. matogrossensis* were not revealed to be monophyletic in one clade). Even when the species are geographically apart, the triafestins conserve some similarities that are confirmed in the two amino acid motifs of these clades, QTAYFTVRCKR with an *E*-value of 1.9 × 10^−118^, and PLTFICTQKGP with and *E*-value of 2.2 × 10^−99^ (Figure S3 and Table S3).

#### 2.3.4. Triatin Subfamily

**Figure 11:**
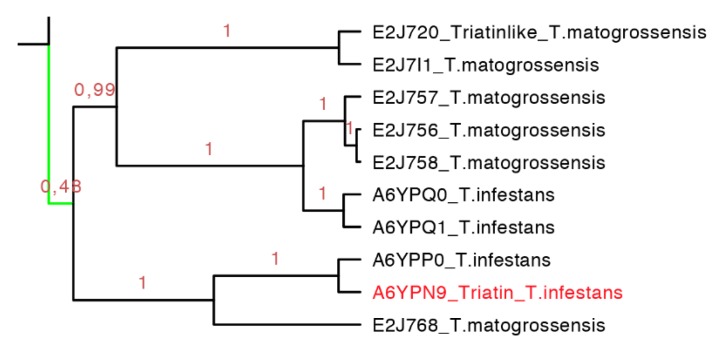
Phylogenetic tree of triatin-like sequences (expanded from the light green clade from Figure 7). In red is shown the only protein that has been functionally characterized.

Triatin is a sequence identified in the salivary gland of *T. infestans* (DQ126070.1), which is a thrombin inhibitor according to Assumpação and collaborators (2011) [41,42]. This monophyletic clade compiled 10 protein sequences (155–181 residues long) whereby there is little knowledge concerning its biological function, and only two species of South America have similar amino acid sequences in this clade (Figure 11). Similar to the triafestin subfamily, the triatins from each species were not revealed to be monophyletic, which means that more than one type of triatins are present in the saliva of one species (e.g., three types of triatins in *T. matogrossensis* are grouped into different clades (Figure 11). The motif found in such protein sequences was SEEIQQ with an *E*-value of 3.2 × 10^−5^ (Figure S4 and Table S3).

#### 2.3.5. Dipetalodipin and Pallidipin

Dipetalodipin is a 165-residue protein identified in the saliva of *D. maxima* that can interact with U-46619 (0.46 µM), which is an analog of the endoperoxide prostaglandin and acts as a thromboxane A_2_ receptor agonist, and arachidonic acid (0.1 mM) with 0.3 µM to inhibit the platelet aggregation. It can also interact with prostaglandin *F*_2α_ (1 µM) or 15(S)-HETE (0.2 µM) with 1 and 3 µM of dipetalodipin, respectively [43]. Pallidipin is a 165-residue protein identified from *T. pallidipennis* that inhibits the platelet aggregation in a dose dependent manner from 20 to 250 nM. Here, it is important to mention that the adhesion of collagen did not affect platelet aggregation; instead the secretion of ATP by the platelet was inhibited at the same rate as the platelet aggregation [44].

**Figure 12:**
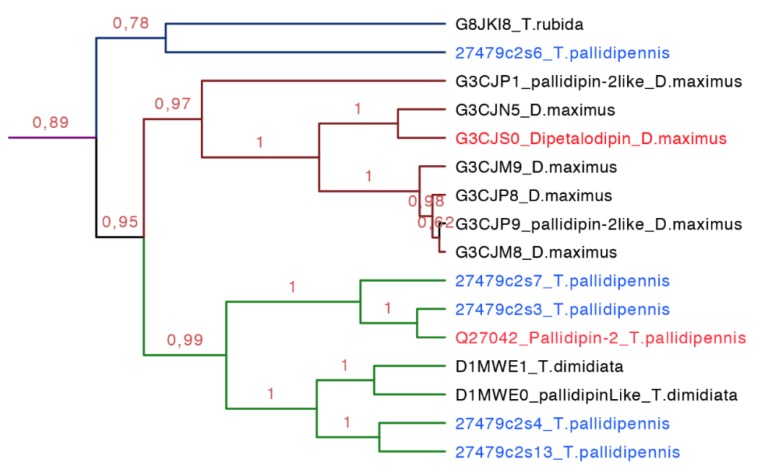
Phylogenetic tree of pallidipin and dipetalodipin (purple clade in Figure 7, expanded). These proteins are structurally and functionally diverse and form three subgroups, one for pallidipin (green), another for dipetalodipin (dark red), and the last one for unknown sequences (dark blue). In red is shown the proteins that have been functionally characterized, and in blue is shown the new proteins reported in this work from *T. pallidipennis*.

In this set of 16 protein sequences (137–232 residues long), proteins with different biological functions were to found share 56% identity. According to our phylogenetic tree (purple clade in Figure 7, expanded in Figure 12), this clade is quite diverse and their protein members have more than one biological activity. They share a lot of amino acid segments in their protein sequence but a specific sequence motif was not found for this clade (Figure S5 and Table S3). So far, dipetalodipins were only from the saliva of *D. maximus*; whereas the pallidipins were from the saliva of two species: *T. pallidipennis* and *T. dimidiata*. The pallidipins were not grouped by species suggesting that there is more than one type of pallidipins per species; for example, there are two different clades of pallidipins from the saliva of *T. pallidipennis*.

#### 2.3.6. Triplatin and Infestilin Subfamily

Triplatin-1 (182 residues long) and triplatin-2 (178 residues long) were identified in the salivary glands of *T. infestans*. They inhibit the platelet aggregation collagen-induced in a dose-dependent manner from 146 to 1463 nM showing that Triplatin-1 has better recognition and inhibits platelet activation via glycoprotein VI (GPVI), but not by the integrin α_2_β_1_ in doses from 112 to 1119 nM [45]. However, Ma and collaborators in 2012 mentioned that triplatin interacts with TA_2_ (U46619, 0.34 µM) and the arachidonic acid (0.16 mM), but cannot be attenuated at higher concentrations. Triplatin can also bind to PGF_2α_ and PGJ_2_ in a stronger manner in doses lower than 2 µM of triplatin, and it was demonstrated that triplatin induces relaxation in vessels. Also, it is mentioned that triplatin does not bind to GPVI [46]. Infestilin is a protein of 151-residues found in *T. infestans* that also inhibits platelet aggregation (AAZ38958).

**Figure 13:**
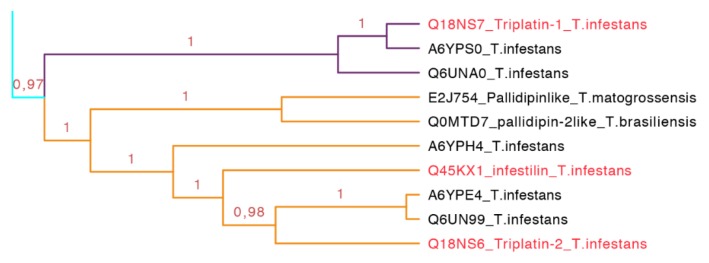
Phylogenetic tree of triplatin and infestilin (cyan clade in Figure 7 expanded). Triplatin-1 (purple) forms a subgroup. Infestin and triplatin-2 (orange) form another subgroup. These proteins seem to be exclusively from South American species. In red is shown the proteins that have been functionally characterized.

This subfamily is represented for species from South America exclusively (cyan clade in Figure 7 expanded in Figure 13). The two types of proteins embedded in this clade, triplatin and infestilin, have high identities: Triplatin-2 and infestilin share 70% identity, triplatin-1 and triplatin-2 share 54% identity but triplatin-1 and infestilin share 49% identity, highlighting the difference between this two subgroups. This set of 10 protein sequences (133–164 residues) had one specific motif IKDRTLPTNVEVTCT with an *E*-value of 1.1 × 10^−4^ (Figure S6 and Table S3).

#### 2.3.7. Dimiconin and Triabin Subfamily

Dimiconin is a 196 residue long protein obtained from the saliva of *T. dimidiata* and it is similar to triabin sharing 62% identity. Dimiconin inhibits the intrinsic pathway of the blood coagulation in a dose dependent manner from 5.6 to 45 µmol/L. It affects the activity of FXa and FIXa, and also the activity of FXII before it is activated [47].

Triabin is a 142 residue long protein identified from the saliva of *T. pallidipennis*. It inhibits thrombin at a molar ratio of 1:1 preventing platelet aggregation and clotting formation at concentrations of 22 nM. Triabin does not affect the amidolytic activity of the thrombin, suggesting that the interaction between triabin and thrombin takes place outside of the activity site. The interaction of triabin with thrombin has a Ki of 3 pM, but in comparison with huridin (protein found in *Hirudo medicinalis*, *K*i of 20 fM) it is higher [44].

**Figure 14:**
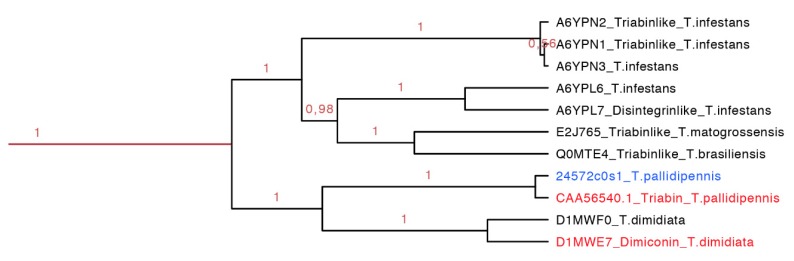
Phylogenetic tree of the triabins and dimiconins (red clade in Figure 7 expanded). It is clear that species from Central and South America share this particular clade, and they have proteins with different functions. In red is shown the proteins that have been functionally characterized, and in blue is shown a new protein reported in this work from *T. pallidipennis*.

The dimiconin and triabin clade was composed of 11 protein sequences (142–172 residues long, red clade in Figure 14 expanded in Figure 7), and it is also separated in two marked clades that show geographic separation. The first clade represents South American species and the second North American species (Figure 14). This clade compiled sequences of two different functions, dimiconin and triabin, that share 60% identity and show a high similarity between sequences, obtaining a total of three motifs for this set. The specific characteristic of this set of proteins is that the position of the fifth cysteine is shifted by two positions in respect to all other triabins, being the only group that presents this peculiarity. The first motif for this clade is FALICRSITFA with an *E*-value of 1.4 × 10^−40^; the two motifs are GGKKNKEQYSFKCKS with an *E*-value of 3.4 × 10^−49^ and SKGITGKFQCD with an *E*-value of 2.6 × 10^−17^ (Figure S7 and Table S3).

#### 2.3.8. Procalin Subfamily

Procalin is a 169 residue long protein identified in *T. protracta* allergen isolated with serum of allergic patients and shows an epitope for immunoglobulin E (IgE) [48]. This interaction could lead to the release of effectors of allergic inflammation mediated by mast cells and basophils. These protein sequences were found in other members of the Triatominae family like *T. infestans*, *T. brasiliensis*, *R. prolixus*, *T. dimidiate* and *D. maxima* but some of them do not have the epitope for IgE [49].

**Figure 15:**
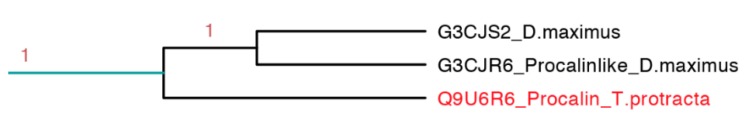
Phylogenetic tree of the only three sequences apparently similar to the only allergenic triabin reported (light blue clade in Figure 7 expanded). In red is shown the only protein that has been functionally characterized.

This clade is the smallest one, represented only by three protein sequences, in which it embeds the only sequence reported from *T. protracta* (procalin). This clade is the only clade with activity reported exclusively from species of North America (Figure 15). Interestingly, this subfamily is closely related to two clades with unknown function and conformed by sequences from two different species (*D. maxima* and *T. rubrida*), which may suggest a phylogenetic affiliation and possible similar functions between these clades (Figures S8–S10 and Table S3).

#### 2.3.9. Clades with Unknown Biological Activities

There are still many proteins with unknown functions in the saliva of the Triatominae; therefore, more than 70% of the clades do not share any similarity with any of the biochemically characterized proteins. This fact suggests that there could be various additional biochemical functions to elucidate, or that some of those clades related to subfamilies with known function could have similar functions. There are five clades that apparently are related species and that no other species share similarity with (Figure 7 clades in gray); three of which were apparently developed from recent evolutionary events according to this phylogenetic analysis (*D. maxima*, *T. rubida* and *T. matogrossensis*, see Figures S8–S10 and Table S3).

## 3. Experimental Section

### 3.1. RNA Isolation, Sequencing and Transcript Annotation

The salivary glands were extracted manually from 7 individuals of *T. pallidipennis* and they were conserved in RNAlater (QIAGEN, Valencia, CA, USA). The RNA extraction was performed following the protocol of the High Pure RNA Isolation Kit (ROCHE, Indianapolis, IN, USA). Poly- adenylated (Poly(A)+) RNA was purified from 10 ng of total RNA, fragmented at a length of 100–500 bases, reverse-transcribed to cDNA, adaptor-ligated and paired-end sequenced on a Genome Analyzer II (Illumina, San Diego, CA, USA), obtaining paired-end reads with a length of 75 bp each. The resulting data passed a quality control (QC) where sequencing adapters and reads of low quality (<30 PHRED) were removed. Transcript reconstruction was performed using the Trinity assembler (rev 25 February 2013) [50] using default parameters, from a pool of reads. Reconstructed transcripts were annotated using the Trinotate pipeline (http://trinotate.sourceforge.net/), a blastx search against the NEMBASE4 (http://www.nematodes.org/nembase4/). Proteins were then conceptually translated from the predicted coding domains of individual cDNA sequences.

### 3.2. Protein Extraction, HPLC and Mass Spectrometry

The saliva from reduviids was extracted using a gentle stimulation of their abdomens. The saliva was collected directly by capillarity from their proboscis, vacuum dried and stored a −20 °C for further use. The saliva (240 µg) was separated using an analytical C_8_ reverse phase HPLC column coupled to an Agilent system (Santa Clara, CA, USA). The components of the saliva were eluted from the column using a gradient of 0%–60% of Acetonitrile in 0.1% TFA in 60 min. The chromatographic fractions were collected manually and analyzed by SDS-PAGE (12.5%) and MS/MS.

The mass spectrometric analysis was performed in an AB SCIEX 3200 QTRAP^®^ LC/MS/MS System. The MS/MS samples were analyzed using Mascot (Matrix Science, London, UK; version 2.3.02). Mascot was set up to search the Massive sequencing RNA of *T. pallidipennis* as a database, assuming a trypsin digestion. The protein search was performed using a fragment ion mass tolerance of 0.52 Da and a parent ion tolerance of 10.0 ppm. Carbamidomethyl of cysteine was specified as a fixed modification, and the oxidation of methionine was specified as a variable modification.

## 4. Conclusions

Forty-two triabin-like proteins were obtained from the salivary glands of *T. pallidipennis*. Five of them belong to the pallidipin and dipetalodipin clade, the other five belong to the triafestin clade and one of them to the triabin clade. Thirty-one protein sequences were not assigned to the remaining five clades; so they are considered proteins without a specific activity. Such a number of triabin-like proteins without a function is similar to that found for the total number of proteins used in this analysis; that is, 70% of the protein sequences had an uncharacterized biological role suggesting that there are a considerable number of protein functions yet to be discovered.

Triabins were recovered distinctively from lipocalins in our phylogenetic analysis with the highest support value, suggesting that their 3D structure similarities reflect a close evolution as members of the calycin superfamily. Here, triabins included the sequences of the German cockroach, the amoeba lipocalin, the nitrophorins and the subgroup with the amine binding proteins characterized for *R. prolixus.* Both NPs and ABPs show protein sequence similarities for triabins; however, our results showed that nitrophorins should be regarded as a subgroup of triabins, because of the weak posterior probability that separate them.

The phylogenetic tree showed 33 different clades and only eight of them were assigned with known biological functions. Therefore, the rest of the clades show that there are still many unknown proteins in the saliva of the Triatominae family. An increase in the knowledge of biological functions of Triatominae saliva proteins within the subfamilies and of the different Triatominae species will surely improve posterior probability values in order to establish better triabin groups. In addition, the sequences available in the databases only came from nine out of 140 triatomine species, and from these protein sequences, the species from North America are poorly studied. Therefore, the saliva of triatomines constitutes a great number of proteins to elucidate in terms of structure and function.

## Supplementary Materials

The following are available online at www.mdpi.com/2072-6651/8/2/44/s1.
