# Supplementary material for: An Insight into the Triabin Protein Family of American Hematophagous Reduviids: Functional, Structural and Phylogenetic Analysis
Source: Toxins (Basel). 2016 Feb 15;8(2):44. doi: 10.3390/toxins8020044 (PMC4773797; doi:10.3390/toxins8020044)
Supplement: Supplementary file 1 [file toxins-08-00044-s001.pdf]

Supplementary Materials: An Insight into the Triabin Protein Family of American Hematophagous Reduviids: Functional, Structural and Phylogenetic Analysis

María J. Hernández-Vargas, Carlos E. Santibáñez-López and Gerardo Corzo

Table S1. Primary structures of proteins obtained from the transcriptome analysis of the salivary glands from *T. pallidipennis*.

ID	Protein Sequences
Q27042	EECELMPPGDNFDLEKYFSIPHVYVTHSRNGPKEQVCREYKTTKNSDGTTTTLVTSDYKT<br>GGKPYHSELKCTNTPKSGVKGQFSVECEVPNGNGGKKKIHVETSVIATDYKNYALLQSC<br>TKTESGIADDVLLLQTKKEGVDPGVTSVLKSVNWSLDDWFSRSKVNCDNMK *
CAA56540.1	AEGDDCSIEKAMGDFKPEEFFNGTWYLAHGPGVTSPAVCQKFTTSGSKGFTQIVEIGYNK<br>FESNVKFQCNQVDNKNGEQYSFKCKSSDNTEFEADFTFISVSYDNFALVCRSITFTSQPKE<br>DDYLVLERTKSDTDPDAKEIC *
22480c0seq1	PASPGIDKCRVETPASNFASSKFFTGTWYVTQAKNISTSVCHQFSTTLAGTTINVKADGFY<br>EIGRKRSFYNVPCSGQKSENGKFTLTCQPKRSDSTTRSTITVQVEVTVMDTDNNKYAVIYR<br>CATSGPHKDDNYLVLQRNKDEVISNLNTILKNKGVSGKLITRKESTHVCPQAQ *
26220c3seq6	QSVQTCTTPTPVMDNFKAPKFHNGTWYVTHVKYVTIPTECRTLTTWQEGDMSFVEHEF<br>TKDGLKGNLRCEAKAEAAKRLSFTCTFNGESIDKTIFIVMASDYNDYALYYICSAITGAN<br>ADRKPGDTVENYLVARRTSGNTEIPAQLQSLTNGMNLQKCS *
26220c3s4.2	QSVQTCTTPTPVMDNFKAPKFHNGTWYVTHVKYVTIPTECRTLTTWQEGDMSFVQHDF<br>TKDGKDGNLRCEAQAEAEKRLSFTCKFNQESIDKTIFIVMDTDYSDYALYYICSAITGAN<br>ADRKAGQIVDNYLIARRTSGNTEIPDKLKSLTEGMNLQKCS *
25921c0s2	QVRSGQCQNYSPMQNFDPSQFFLGSWFVTHAKNGPEFAACRTYQTSVNGKNINFNGD<br>GYYGDKQKNSYYQVRCTGPKNSGGTGKFSLSCSLQSLSNSATRNQINIASNLELTIIKTDY<br>SQYAIVYRCAKYSTVQVTKDNLMVLHRDQNSLAPNIANTFQQATSMPLSGLPFTRQSTT<br>CKKFDNHITDDLIDELLKY *
25921c0s1.4	QVRSGQCQNYSPMQNFDPSQFFLGSWFVTHAKNGPEFSVCRTYQTKVSGRDINFNADG<br>YYGDTKANSYYKVRCTGPKDSGKKGRFSLSCNLNSLSNSASPTQKSITFNLDLTIIKTDYS<br>QYAIVYRCAWYSTLQVTTDNLMVLHRDKNALAPNIANNFQQATRMSLSGLPYTRQRTT<br>CKQMDNHITDDLIDELLKY
26887c0s1.4	SYQPYISEKCQDVKAMSNFQPTRFFSGTWYVTHAKNGTAATVCHKYKTKKEQNGKFSF<br>DYGYYNNGNEEPFFQVHCAETKRIKNKPFSFYCKLIKGQESSNFKQYNVDLTFIGTDYESFAI<br>FYRCVPIGTLGYADNFLVLHRTKAISSTYAKAKNVLEKQGLFLDSFLNRKNSNCKNNPKF *
27479c2s12	WYVTNAKHGSNSTVCREYNGKRENGNPVLNGDGYYSHGNLKIYFEVRCDKQSDTNYK<br>LTFSCTQKGPAGTNMNFQFQLEVTVLSTDYDDFAIMYRCVKFPPQLGSRIEDNVLVLHR<br>KDCKTEDKDRVEETLKKNGWSLDSFKSRKGVNCPEPPQK *
27479c2s11.5	WYVTNAKHGSDRTVCREYKGKRQNGIRQLIADGYYSYGQDQTAYFNVLCHKQSDRNY<br>KLTFNCTQRGSITDDQKVIIQFQLEITVLLTDYDDFAIMYRCVKFPPQLGSRIEDNVLVLRR<br>NPNTDNKNPLIETTLKSQDLSLDTFKSRKGVTCPKLPQK *
27479c2s8.5	WYVTNAKHGSNSTVCREYNGKRENGNPVLNGDGYYSFRNLKIYFEVRCDKQSDTNYKL<br>TFSCTQKGPAGTNMNFQFQLEVTVLSTDYDDFAIMYRCVKFPPQLGSRIEDNVLVLHRD<br>ATKTNDKNPQIEDILKKQDWSLDTFNSRDGVECPLPPQK *
27479c2s9.5	WYVTNAKHGSNSTVCREYKGKRENGNPVLNGDGYYSVGSQKIYFEVRCDKQSDKNYKL<br>TFSCTQKGPAGSKMNFQFQLEVTVLSTDYDDFAIMYRCVKFPPQLGSRIEDNVLVLRRNP<br>NTDNKNPLIETTLKSQDLSLDTFKSRKGVTCPKLPQK *
27479c2s14	WYVTNAKHGSDRTVCREYKGKRQNGIRQLIADGYYSYGQDQTAYFNVLCHKQSDRNY<br>KLTFNCTQRGSITDDQKVIIQFQLEITVLLTDYDDFAILYRCGKIPSSSGSRIEDNVLVLHRK<br>DCKTEDKDRVEETLKKNGWSLDSFKSRKGVNCPEPPQK *
21634c0s2	QSRLGAKQDLIIQDFFKGSWFVTHIDGVNEATCREYITKIENDAIKLNAVGEHKLTKEQE<br>KNSTTYCSSSKGKALKPEGPFVLQCRHIYGDNERTTFFGLTFSVIETDYTDYALVHRCTKY<br>NNMNFITGNLLLLHRSKTSDGSKATTSLEKHSLSLQQFQKTGC *

21634c0s1.4	QSRLGAKQDLIIQDFFKGSWFVTHIDGVNEATCREYKSNIENGTIKLNAFGEHKLFEQYE<br>YSTTNCSSTKGKQLNQAGPFALQCRHTYGDNDYTYNNYYGLSFSVIETDYTDYALVHRC<br>TKYNNMNFITGNLLLLHRSKTSDGSKATTSLEKHSLSLQQFQKTGC *
22866c0s1	ATKFFSGTWYVTHVQKVPSPNVCHTFAVNQKDGKFIFGLGKDGKENQVQCESKREEDK<br>ITFSCQSVTGNFTFQSVFIVMGASYDDYAVLYNCVTVSRFKADNYVVLSRKSDKEEIPAEA<br>KSLTNELNLRKCTDIRKSVN *
26508c1s3	SQCQNTLRTKQDLNIQSFFKGSWYVTHIKDGGDEASCREYKTSLDGIQNLIKLDADGQY<br>KFKGQTKFYTTKCSSVSGTPLNPPTGKYVLKCRHTYGGTENIFFDLKLSIIETDYSNYALVY<br>RCTKYDDSSLNLKYGNLLLLQRSKTANGSKATASLQKNQSSLKQFKKTA *
26508c1s1.3	QKAKCQSDLEAKKDLDIQKFFKGSWYVTHIKDGGDEASCREYKTSLDGIQNLIKLDADG<br>QYKFKGQTKFYTTKCSSVSGTPLNPPTGKYVLKCRHTYGGTENIFFDLMLSVIETDYSNYA<br>LVYRCTKYDDPSLNLQYGTLLLLHRSKTSDGSKATTSLQKHRLSLQQFKKTAGC *
26220c3s3	QSVQTCTTPTPVMDNFKAPKFHNGTWYVTHVKYVTIPTECRTLTTSKDGNKYIVEHDFT<br>KNGVKGRLHCEASPEAEKKLTFTCTFNGESIDKTIFIVMDTDYSDYALYYICSAITGANAD<br>RKPGDTVDNYLVARRTSGNTEIPDKLKSLTGEMDFKKCT *
26220c3s5	EVGSLSECENPTAMEGFSATKFHSGTWYVTHVTKVTEPTECRTLTTSKDGNKYIVEHDFT<br>KNGVKGRLHCEASPEAEKKLTFTCTFNGESIDKTIFIVMDTDYSDYALYYICSAITGANAD<br>RKAGQIVDNYLIARRTSGNTEIPDKLKSLTEGMNLQKCS *
27168c1s1	EEPSNNNECNRAAMTNFDSTRYLQINRAFVTHSREGTGAIICRLYKTQKSGDKTDEVDIN<br>IYEYFEKRRDIYYSETHCNTTLARIEKGTFVSSCKEVMLTARRSDTTMKPKPRIIEVYTSVID<br>TDYDKYIIHYRCVKTKSKIIDNIEVLQTNKNAGDEPIKQALEKNGLELEDFIAHGNKPEEC<br>ENIDNGKKELLL *
27168c1s14	YDAMANFDSTKYLQIAPLYSTYEKNAKYFTYCRVYKSNTKSDDKINMNIWGYYQESEKN<br>HYYEIVCSTKEQNFKTGQYWAECNVVKDSNYEDTSISEPFKLYMSVIDTDYDNYAILYLCI<br>SDELGFEDNVEVLQKNP *
27168c1s3	EEPSKTCNYQAMANFDSTKYLQIAPLYSTYEKNAKYFTYCRVYKSNTKSDDKINMNIWG<br>YYQESEKNHYYEIVCSTKEQNFKTGQYWAECNIVKDTHYTEPIKPYQLYLSVIDTDYQNY<br>AILYNCNSNVTHFRDNIEVLQKDPGTDDNLVKAALETKGMNLENFIQRNFTTVCQNEN<br>KKKEEK *
27168c1s8	EEPSNNNECNRAAMTNFDSTRYLQMTPLYSTYAMYGNLTVCRVFQSITKSGDEVNINIH<br>GYYQESGQIYNYEMSCNTNEENFKTGQYRAECNVKDTHYTEPIKPYQVYLSVIDTDYQN<br>YAILYNCNSNETHFRANIEVLQKDPGTDDNLVKAALETKDMKLEKFIQRNFTTVCQNEN<br>KKKEEK *
27168c1s10.6	TPNNKKCGYKPMENFDSTRYLQITTPLYLTHTKLESNYSVCREFKSIKKSGDTVDINIHGYY<br>QDSGKIYNYELLCSTAEESFNKGQYLSVCEILKDTHDEEPLTSMSFEQHMSVIDTDYDSYAI<br>LYTCFNDESGFEENVVVLKKDPGGSDEAVTTALKNHEMDLKKFFPWNYAYCQNHKA *
27168c1s12.6	EQTYNPEYPSNNLECNNQAMANFDSTKYLQTPLYSTYARDSLNITVCRAFKPITKSGDQV<br>NININGYYQDSGKIYNYELLCSTAEESFNKGQYLSVCEILKDTHDEEPLTSMSFEQHMSVI<br>DTDYDSYAILYTCFNDESGFEENVVVLKKDPGGSDEAVTTALKNHEMDLKKFFPWNYA<br>YCQNHKA *
27168c1s13	EQTYNPEYPSNNLECNNQAMANFDSTKYLQINRAFVTHSREGTGAIICRLYKTQKSGDK<br>TDKININIYEYFEPRRDIYYSETHCNTTLARIEKGTFVSSCKEVMLTARRSDTTMKPKPTIIE<br>VYTSVIDTDYDKYIIHYKCTKTKSGIIDNIEVLQTNKNAGGEPIKQALEKNGLELEDFIAHE<br>NKHKECENIDNGKKELLL *
27479c2s3	EECELTPPEDNFDLEKYFSIPHVYVTHSRNGPKEQVCREYKTTKNSDGTTTTTLVTSDYKT<br>GGKPYHSQLKCTNTPKNGGKGQFSVECEVPNGNGGKKKIHVETSVIATDYKNYALLQS<br>CTKTESGIADDVLLLQTKKEGVDPGVTSVLKSVNWSLDKWISRTEVNCDNIQN *
27479c2s7<br>**(Fraction 22)**	KECELMPPASNFDSEKYFDIPHVYVTHSRNGPKEQVCREYNTTKIQGDTTPYTVVTSDYKI<br>RGETHHSQLKCTNTPKNGGKGQFSVECEISNGNGGNKKKVQFETSVFATDYKNYALLQ<br>SCTKTESGIADDVLLLQTKKEGVDPGVTSVLKSVNWSLDDWFSRSKVNCDNMK *
25439c1s1	EKCTLLPAMDKFDSDRYFSVLHLFVTHSKNEPKQQVCREYETIKKKNRDGTSTTRMLEVY<br>KIGGKQHKTVLDCINTPKSGSVGQFSVDCQVKVAENVSTKNKNQLEISIIATDYKN *
27479c2s4	KECELMPPASNFDSEKYFDIPHVYVTHSRNGPKENVCREYKTTKSTGGLTLTDVIVNTGG<br>KQRTVLNCINTVKNIKPGHYFLECEAPNGDNQKIQLE *
27479c2s13	KECELMPPASNFDSEKYFDIPHVYVTHSRNGPKENVCREYKTTKNTDGTTHTEVIVKTGG<br>KQRTVLNCINTQKNGKPGQYSVECEVPNGNGGNQKIQLETSVFATDNEKYALLQSCNK<br>DGESEDDIFVLQTNKDPVEQGVTSVLKSVNWSLNDWFSRSKVNCDNMK *

27479c2s6	KECQLMPATDDYNADQYFSIPRVYAIYSKNGPEENVCREYETKKKTDGTIVTTVSGDKK<br>NRGQQQTVLACTNREKSGSKGQFFVECEVPGKNGGNMKIQVETSVLATDN *
24967c0s3	FEAPKYHTGKWYVTHVKESPNDCQILRTSQNGETSIVEHDFIHNGKSGQQHCEALPETE<br>KRLTFTCMFGDVSIDKTIQIVMATDYENYSLYYLCSTFASGPDKGKTVDNYLLASRKNID<br>AEIPDKLK *
24967c0s1.5	CTIPTKVMDNYQAPKYHTGEWYVTHVQKSPNDCQILTTSQDGDTSIVEHDFTYNGKAG<br>KQHCEALPETEKRLTFTCMFGDVSIDKTIQIVMATDYENYSLYYLCSTFASGPDKGKTVD<br>NYLLASRKGITAEIPKELESLTSKLNLQKCNYS *
26220c3s7	CAYSQFVQQCATPTTVMQNFEAPKFHNGTWYVTHVKYVTIPTECRTLTTSKDGNKYIVE<br>HDFTKNGVKGRLHCEASPEAEKKLTFTCTFNGESIDKTIFIVTDTDYDDYALYYICSAITG<br>ANADRKPGDTVENYLVARRTSGNTEIPAQLQSLTNGMNLQKCS *
24967c0s2	CTIPTKVMDNYQAPKYHTGEWYVTHVQKSPNDCQILTTSQDGDTSIVEHDFIHNGKSGQ<br>QHCEALPETEKRLTFTCMFGDESIDKTVQIVMDTDYNDYSLYYLCSTFTSGPNKGKTVDN<br>YLLASRKNIDAEIPDKLK *
21465c0s1	FAFPEYPPDTTECLDFEDYENFNTAKFLKGIWYVTHARYGSNSTVCREYKTRLRKNGIINI<br>VADGYYNFGGQPRYYRVRCEGTKEEENGKLSLNCKQQSRGKVNKIIFNYQLDLSVIETDY<br>DKFAVIYTCAKFPASNELSVEDNILILHRDKDNDNSGVETILQQHESSLQEFLSRKDSTCL<br>PSPVKN *
24572c0s1	AEGDDCSLEKAMGDFKPEEFFNGTWYLAHGPDVTSPAVCQKFTTSGSKGFTQIVEIGYN<br>KFESNVKFQCNQVDNKNDEQYSFKCKGSDNTEFEADFTFISVSYDNFALVCRSITFTSQPK<br>EDDYLVLERTKSDTDPDANEIC *
24833c0s3	DTIKYGGKSCQPIPTMENFDAARFFSGSWSLTHSTPSARVTPSTICRDYELKVHENGTFGV<br>TYGYFENSGRTNRYDIHCIGTKSNQPGLSSFDCYLTNARGEKTHTHIDAYFIAADYENYC<br>LVYRCVTSDATFEDNVFLLYRNKDYIPSDEEVKNIIQPFGLGLEQFISRKDATCTNK *
24833c0s1<br>**(Fraction 22, 23 and 24)**	DTIKYGGQTCQQQVPIMKDFDVERFFSGSWSLTHSTRSPRVTESTICRDYELKVHENGTFG<br>VTYGYFENSGRNNRYDINCLGTRSDQPGLSYFDCYLTNARGEKTHTRIDGYFVTTDYDN<br>YCLVYRCVTSDDKFEDNVFVLYRNKNYIPKDEEVKKIIEPYGLGLEQFISRKDATCTNK *
24833c0s2<br>**(Fraction 22)**	EKLEYGKGVCQNNKLDGLVNLNAQKFFSGTWYLTHATKSTRVTLSTICRDFEPKQKEDG<br>TFEVTYGYYENGGKQNHYDVSCSGTQDKTRLDIFNFDCKSNNERGETTSFHIDGSFLATD<br>YDSYGVVYRCVTTGTLTEDNVFLIHRQKNPSDEEVTKILTHYGLSLGDVISRKDATCTNK *
23242c0s1.5	NFAGIRECQQVTAMNGFDSNKFFRGTWHMTNVQNGLFSIVCQDLETENKNGKLITKYNY<br>NKNGKEHTVQCESNGQGKNGEIPFDCEIKSKRFFKFLNKVTKFQANFKIMTTDYNDYALL<br>YKCVTLKSGTKADNYVVLSRNKNDYKIPGSAQSLLQINGVSLKKCSELVKADDINSEVV *
23242c0s2	GITSINECQKVTAMQNFDSTKFFRGTWYMTNVMNGLFSIVCQELETTKDGAQLFIDYNYN<br>KNGKENYVRCESKGQEKNGQIPFDCKIEKSGLFNFFSKSKKFQANFTIMTTDYDNYALFYK<br>CVTLQSGVKADNYAVLRRSKNDYNIPGSVQSFLQINGVSMKKCSELVNADDINREVI *

Table S2. Amino acid sequence identification (ID) of the species used for the phylogenetic analysis.

Specie	No. Secs	ID
Rhodnius prolixus	33	AAS94228.1, AAC26160.1, 1U0X_A, 4NP1_A, AAB09090.1, AAB41587.1, Q7YSY5, Q7YT08, Q7YSZ9, Q7YT10, Q7YT05, Q7YSY9, Q94732, Q7YT07, Q7YT06, Q7YT02, Q94731, Q7YT03, Q7YT04, Q7YT09, Q7YT14, Q86PT9, Q26239, Q26241, Q7YT13, Q7YT15, Q9TY55, Q94733, Q7YT12, Q7YT11, Q6PQK2, O77000, Q94734
Triatoma matogrossensis	68	A6YPR4, E2J702, E2J703, E2J704, E2J705, E2J706, E2J707, E2J708, E2J709, E2J710, E2J711, E2J712, E2J713, E2J715, E2J716, E2J717, E2J718, E2J720, E2J721, E2J722, E2J723, E2J724, E2J725, E2J726, E2J727, E2J728, E2J729, E2J730, E2J731, E2J732, E2J733, E2J734, E2J735, E2J736, E2J737, E2J739, E2J740,E2J741, E2J743, E2J744, E2J745, E2J746, E2J747, E2J748, E2J749, E2J750, E2J751, E2J752, E2J753, E2J754, E2J755, E2J756, E2J757, E2J758, E2J759, E2J760, E2J762, E2J763, E2J765, E2J766, E2J767, E2J768, E2J769, E2J770, E2J7H8, E2J7H9, E2J7I1, E2J701
Triatoma protracta	1	Q9U6R6
Triatoma rubida	34	G8JKC6, G8JKC7, G8JKC8, G8JKC9, G8JKD0, G8JKD1, G8JKD2, G8JKD3, G8JKD4, G8JKD5, G8JKD6, G8JKD7, G8JKE5, G8JKE6, G8JKF6, G8JKF7, G8JKF8, G8JKF9, G8JKG0, G8JKG2, G8JKG3, G8JKG4, G8JKG5, G8JKG8, G8JKG9, G8JKH3, G8JKH5, G8JKI0, G8JKI1, G8JKI2, G8JKI4, G8JKI5, G8JKI6, G8JKI8
Dipetalogaster maxima	33	G3CJM3, G3CJM4, G3CJM8, G3CJM9, G3CJN0, G3CJN2, G3CJN3, G3CJN4, G3CJN5, G3CJN6, G3CJN7, G3CJN8, G3CJP0, G3CJP1, G3CJP4, G3CJP5, G3CJP6, G3CJP8, G3CJP9, G3CJQ2, G3CJQ3, G3CJQ4, G3CJQ5, G3CJQ6, G3CJR4, G3CJR5, G3CJR6, G3CJR7, G3CJR8, G3CJS0, G3CJS2, G3CJS5, G8JKH9
Triatoma infestans	60	A6YPC8, A6YPC9, A6YPD5, A6YPD6, A6YPE4, A6YPE5, A6YPE6, A6YPE8, A6YPE9, A6YPF0, A6YPF2, A6YPF4, A6YPF6, A6YPF8, A6YPG0, A6YPG3, A6YPG6, A6YPH0, A6YPH2, A6YPH4, A6YPH5, A6YPH8, A6YPI0, A6YPI6, A6YPK7, A6YPL3, A6YPL6, A6YPL7, A6YPM0, A6YPM1, A6YPM4, A6YPN1, A6YPN2, A6YPN3, A6YPN6, A6YPN9, A6YPP0, A6YPP6, A6YPP8, A6YPQ0, A6YPQ1, A6YPR3, A6YPR5, A6YPR6, A6YPR8, A6YPR9, A6YPS0, A6YPS5, A6YPS9, A6YPT2, A6YPU1, A7BJ45, A7BJ46, Q18NS6, Q18NS7, Q45KX1, Q6UN98, Q6UN99, Q6UNA0, Q6UNA1
Triatoma brasiliensis	23	A0MK83, A0MK85, A0MK87, A0MK88, A0MK92, A0MK94, Q0MTC5, Q0MTC6, Q0MTC9, Q0MTD0, Q0MTD2, Q0MTD3, Q0MTD4, Q0MTD7, Q0MTD8, Q0MTD9, Q0MTE1, Q0MTE3, Q0MTE4, Q0MTE7, Q0MTE8, Q0MTE9, Q0MTF1
Triatoma pallidipennis	44	See Table 1
Triatoma dimidiata	43	D1MWA7, D1MWA8, D1MWA9, D1MWB0, D1MWB1, D1MWB2, D1MWB3, D1MWB4, D1MWB5, D1MWB6, D1MWB7, D1MWB8, D1MWB9, D1MWC0, D1MWC2, D1MWC3, D1MWC4, D1MWC6, D1MWC7, D1MWC8, D1MWC9, D1MWD0, D1MWD1, D1MWD2, D1MWD3, D1MWD4, D1MWD5, D1MWD6, D1MWD8, D1MWD9, D1MWE0, D1MWE1, D1MWE2, D1MWE3, D1MWE4, D1MWE5, D1MWE6, D1MWE7, D1MWF0, D1MWF1, D1MWF2, D1MWF4, D1MX91
Blattella germanica	6	A9XFW8, B7TYB1, B7TYB2, C3RWZ4, C3RWZ5, P54962
Naegleria gruberi	1	D2UZV1
Human	3	gi|313856, gi|7245434, gi|308387777

Table S3. Amino acid sequences of proteins of each representative clade.

ID	Amino Acid Sequences
	Nitrophorin and amine binding protein subfamily
>AAS94228.1_NP-7	--LPGECSVNVIPKKNLDKAKFFSG-TWYETHYLDMDP--QATEKFCFSFAPRES-GGTV<br>KEALYHFNVDSKVSFYNTGTGPL---ESNGAKYTAKFNTVDKKGKEIKPADEKYSYTVTV<br>IEAA-KQSALIHICLQEDGKDIGDLYSVLNRNKN—ALPNKKIKKALNKVSLVLTKFVVT<br>KDL--DCKYDDKFLSSWQK---------
>Q6PQK2_Nitro	---PGECSVNVIPKKNLDKAKFFSG-TWYETHYLDMDP--QATEKFCFSFAPRES-GGTV<br>KEALYHFNVDSKVSFYNTGTGPL---ESNGAKYTAKFNTVDKKGKEIKPADEKYSYTVTV<br>IEAA-KQSALIHICLQEDGKDIGDLYSVLNRNKN--ALPNKKIKKALNKVSLVLTKFVVT<br>KDL--DCKYDDKFLSSWQK---------
>Q7YT13_Nitro	---SEDCSVNIIPKQRLAKAKFFSG-TWYETHYLDTNP--EVTDKFRFSFAPRQS-GGTV<br>KEAFYHFSSK--<br>--<br>----------------------------
>AAC26160.1_NP-3	-----DCSTNISPKKGLDKAKYFSG-TWYVTHYLDKDP--QVTDPYCSSFTPKES-GGTV<br>KEALYHFNSKKKTSFYNIGEGKL---GSAGVQYTAKYNTVDKKRKEIEPADPKDSYTLTV<br>LEAD-DSSALVHICLREGPKDLGDLYTVLSHQKT--GEPSATVKNAVAQAGLKLNDFVDT<br>KTL--SCTYDDQFTSM------------
>O77000_Nitro	---SGDCSTNISPKKGLDKAKYFSG-TWYVTHYLDKDP--QVTDPYCSSFTPKES-GGTV<br>KEALYHFNSKKKTSFYNIGEGKL---GSAGVQYTAKYNTVDKKRKEIEPADPKDSYTLTV<br>LEAD-DSSALVHICLREGPKDLGDLYTVLSHQKT--GEPSATVKNAVAQAGLKLNDFVDT<br>KTL--SCTYDDQFTSM------------
>Q94733_Nitro	---SGDCSTNISPKKGLDKAKYFSG-TWYVTHYLDKDP--QVTDPYCSSFTPKES-GGTV<br>KEALYHFNSKKKTSFYNIGEGKL---GSSGVQYTAKYNTVDKKRKEIEPADPKDSYTLTV<br>LEAD-DSSALVHICLREGPKDLGDLYTVLSHQKT--GEPSATVKNAVAQAGLKLNDFVDT<br>KTL--SCTYDDQFTSM------------
>Q9TY55_Nitro	---SGDCSTNISPKKGLDKAKYFSG-TWYVTHYLDKDP--QVTDPYCSSFTPKES-GGTV<br>KEALYHFNSKKKTSFYNIGEGKL---GSPGVQYTAKYNTVDKKRKEIEPADPKDSYTLTV<br>LEAD-DSSALVHICLREGPKDLGDLYTVLSHQKT--GEPSATVKNAVAQAGLKLNDFVDT<br>KTL--SCTYDDQFTSM------------

>Q26241_NitroNP-2	-----DCSTNISPKQGLDKAKYFSG-KWYVTHFLDKDP--QVTDQYCSSFTPRES-DGTV<br>KEALYHYNANKKTSFYNIGEGKL---ESSGLQYTAKYKTVDKKKAVLKEADEKNSYTLTV<br>LEAD-DSSALVHICLREGSKDLGDLYTVLTHQKD--AEPSAKVKSAVTQAGLQLSQFVGT<br>KDL--GCQYDDQFTSL------------
>AAB41587.1_NitroProlixin-s	-----DCSTNISPKQGLDKAKYFSG-KWYVTHFLDKDP--QVTDQYCSSFTPRES-DGTV<br>KEALYHYNANKKTSFYNIGEGKL---ESSGLQYTAKYKTVDKKKAVLKEADEKNSYTLTV<br>LEAD-DSSALVHICLREGSKDLGDLYTVLTHQKD--AEPSAKVKSAVTQAGLQLSQFVGT<br>KDL--GCQYDDQFTSL------------
>Q7YT14_Nitro	---SGDCSTNISPKQGLDKAKYFSG-KWYVTHFLDKDP--QVTDQYCSSFTPRES-DGTV<br>KEALYHYNANKKTSFYNIGEGKL---ESSGLQYTAKYKTVEKKKKK-------------<br>----------------------------KKKNFLAPPRAPHKT---------------<br>----------------------------
>1U0X_A_NP-4	-----ACTKNAIAQTGFNKDKYFNGDVWYVTDYLDLEPD-DVPKRYCAALAAGTA-SGKL<br>KEALYHYDPKTQDTFYDVSELQV---ESLG-KYTANFKKVDKNGNVKVAVTAGNYYTFTV<br>MYAD-DSSALIHTCLHKGNKDLGDLYAVLNRNKD--AAAGDKVKSAVSAATLEFSKFIST<br>KEN--NCAYDNDSLKSLLTK--------
>Q94734_Nitro	-----ACTKNAIAQTGFNKDKYFNGDVWYVTDYLDLEPD-DVPKRYCAALAAGTA-SGKL<br>KEALYHYDPKTQDTFYDVSELQV---ESLG-KYTANFKKVDKNGNVKVAVTAGNYYTFTV<br>MYAD-DSSALIHTCLHKGNKDLGDLYAVLNRNKD--AAAGDKVKSAVSAATLEFSKFIST<br>KEN--NCAYDNDSLKSLLTK--------
>Q7YT15_Nitro	---SGKCTQNAIAQTGFKKDQYFNGGVWYVTDYLDLEPD-DVPKRYCAALAAGTA-SGKL<br>KEALYHYDPVSKDTFYDVSELTQ---ESSG-KYTANFKKVDKNGNVKVDVTAGNYYTFTV<br>MYAD-DSSALIHTCLHKGNKDLGDLYAVLNRNKD--AAAGDKVKSAVSAATLEYSKFIST<br>KEN--NCAYDNDSLKSLLTK--------
>4NP1_A_NP1	-----KCTKNALAQTGFNKDKYFNGDVWYVTDYLDLEPD-DVPKRYCAALAAGTA-SGKL<br>KEALYHYDPKTQDTFYDVSELQE---ESPG-KYTANFKKVEKNGNVKVDVTSGNYYTFTV<br>MYAD-DSSALIHTCLHKGNKDLGDLYAVLNRNKD--TNAGDKVKGAVTAASLKFSDFIST<br>KDN--KCEYDNVSLKSLLTK--------
>Q26239_Nitro	---SGKCTKNALAQTGFNKDKYFNGDVWYVTDYLDLEPD-DVPKRYCAALAAGTA-SGKL<br>KEALYHYDPKTQDTFYDVSELQE---ESPG-KYTANFKKVEKNGNVKVDVTSGNYYTFTV<br>MYAD-DSSALIHTCLHKGNKDLGDLYAVLNRNKD--TNAGDKVKGAVTAASLKFSDFIST<br>KDN--KCEYDNVSLKSLLTK--------

>Q86PT9_NitroABP	---ASGCS-TVDTVKDFNKDNFFTG-SWYITHYKLGDSTLEVGDKNCTKFLHQKTADGKI<br>KEVFSNYNPNAKTYSYDISFAKVSDFDGNNGKYTAKNVIVEKDGRKIDE----RTLQVSY<br>IDTDYSKYSVVHVC--DPAAPDYYLYAVQSRTENVKEDVKSKVEAALGKVGLKLSGLFDA<br>TTLGNKCQYDDETLQKLLKQSFPNYEK-
>Q7YT11_Nitro	---ASGCL-TVDTVKDFNKDNFFTG-SWYITHYKLGGGTLQDIDKNCTKFLHKKTNDGKI<br>REVFSNYNPNGGTYSYDISFASVKTFDGNNGKYTAKNVIVNQDGTKIDD----RKLQVSY<br>IDTDYSKYSVVYVC--DPSAPEYYLYAVQSRNENI-NGVKDKVETALGKVNLKLKDLFDA<br>TTL-SSCKYDEDTLKKLWDRSYPEYEKE
>Q7YT12_Nitro	SGADKRCD-NPEPMTPFDVDKFFKD-AWYITHYKFGADTGSNNDKYCTKILQKIE-NDNI<br>KEVFSIDNTTTEAYSYYLSFSKKSSFDTTYGKYTAKHIQVDKVGKELEE----HSITVTY<br>LDTDYDSYSVVYVC-GEIMENLFSLYAVQSRSQTLNQDVETKVKSALNGVNLKLDKLSSI<br>KDF--GCKYDDSTLNALLSKSFTHETK-
>E2J733_Nitro	--VNEECR-NIKTKTDFDPEKYYGR-IWYGIYILFTNVKLTSEDYACLRTKSNFLENGKV<br>REIETVYVPKNEAYAYSESYINAVDLRGGVSKFAAIGRPIDKDGRPLLE--EFYPLQYTI<br>VDTDYDNYAVVYMCAQIPSGQTLSIYSILNRNSGA-KDINGKVLSILDEIGVKLDDFTRI<br>NQN--DCNDRPVVESRRLV---------
>D1MX91_Nitro	---PEECP-NIKTKTDFDPLQNFGK-TWYVTHALFTNVQLTPEDFACLNSKTNLLENGKV<br>KEIETVYVPKNEYYVFTESYLNAADFKGGIAKFTALSRPIDKHERPLMK--QFYPIKINI<br>VDTDYDDYAVVYSRAHAPNGQIISIYTIANRGSGV-KRENETVSSILDEIGVKLDDFTQI<br>NQD--KCPRLTSCWK-------------
	Rhodnius prolixus aggregation inhibitor-1 subfamily
>Q94731	----ANPPKMPTGCKDLNSKAVKDFKYNDFFKDKWILTHAE-RVTHPDACETF--TVNGN<br>-KITFSLG-GKEVSCTLVKVEGAK-FTKFNC-ELQ-GKKFTAY------LSVLATDYKNY<br>ALVYRCGSHESPT-KDNFLVAQRRKQSTFPSALESQVSKVGFGLKKDSFKKFNC-
>AAB09090.1_RPAI-1	----ANPPKMPTGCKDLNSKAVKDFKYNDFFKDKWILTHAE-RVTHPDACETF--TVNGN<br>-KITFSLG-GKEVSCTLVKVEGAK-FTKFNC-ELQ-GKKFTAY------LSVLATDYKNY<br>ALVYRCGSHESPT-KDNFLVAQRRKQSTFPSALESQVSKVGFGLKKDSFKKFNC-
>Q7YSY9_RPAI-7	----ATVPKMPSGCADVHNKAVSDFNFDKFFKGQWHLTHAKLRVTTAKTCETF--TVNGD<br>-ELTFTLN-GQSVSCKLEKVTGAR-FTKFNC-QMG-QAKFTSY------VSVLATDYDNY<br>VLVYRCGSHEGPT-KDNYLVGQRKKVQHFLPD----------------------
>Q7YT10	QY--GGPPKMSRGCKDIYNRGVDNLNYKQFFTGQWFLTHGE---RVSSKCDTV--SVNGD<br>-KITFKLR-GAQINCQLENVPDAK-FTKFNC-KKSVSKTFSTE------ISVLATDNNNY<br>ALVYRCGVLEDDNYKDNTVVMQRQKQAPFPPALESEVGKFGHGLKKDSFKVLNC-

>Q94732	AAGASATTTMPKECLELKGDIKPGFDANQFFTGDWYWTHAR-DPKHPKLCQKY--QATSD<br>LRLKFNGNSGSDVTCQGAKVIGKEGFYSFQC-TTS-GVTFTSF------MAVVETDYNNY<br>ALLYRCGRYGSSAVEDNFLVFNRQSSGGIPGGLTTKLSQLD--LTPTSFTKLGCT
>Q7YT09	----AEVTSIPTGCNALSGKIMSGFDANRFFTGDWYLTHSR-DSEVPVRCEKY--QTGSN<br>LQLNFNGKNG-DVKCSGSTVSGNQGFYSFQCTTTS-GGSFTSY------MAVVETDYANY<br>ALLYRCGLYGSTTPKDNFLLFNRQSSGEIPAGLSTKLNQLE--L--TSLNKLGCS
>Q7YT07_RPAI-6	-----KRVPTPEGCRDVYNEADPNFKLKKFFNGSWYLTHAK-HQNHSVLCTKFGMTMKPL<br>-EIKYEMG-GVNVTCKGTKIKGTR-RTEYVC-EGN-RGTPTPYTNYGATMSVIDTDYTNY<br>ATVYVCKKNGKH--EDNVFVLSRIRTGEPPEAAKQSLQKLR--------------
	Triafestin subfamily
>E2J7H8_triafes-2Like	---IFLGILAFVVADYPP-IEKCSHPSSMAYFNQKKFLAGKWCVTKAKHGSNSTVCRQYK<br>GKFNDEKQQFIGDGYYNFK-CQTTYFTVRCSRVP--NTNVQQPLQFICTQKKP--DQKDI<br>QFQFQLEVTVLDTDYATYAGMYRCVQLPQEL-GSMFEDNTLLIQRNANLEVDEN-KIEKA<br>LR---LSFDSFISRNDVKGGCPELPSK-NKNKKPKT---
>E2J741_triafestinlike	--------------DYPP-IEKCNHPSAMANFNQKKFLSGKWYVTKAKHGSDSTVCRQYK<br>GKFNDEKQQFIGDGYYNFK-CQTAYFTVRCSRVP--NTNVQQPLQFICTQKKP--DQKDI<br>QFQFQLEVTVLDTDYATYAVMYRCVQFPQEL-GSMFEDNTLLIQRNANLEVDEN-KIEKA<br>LR---LSFDSFRSRNDVKGGCPELPSK-NKNKKPKT---
>E2J729_triafestinlike	--------------DYPP-IEKCNHPSAMANFNQKKFLKGKWFVTKAKHGSDSTVCRQYK<br>GKFNNEKQQFIGDGYYNFK-CHTAYFTVRCSRVP--NTNVQQPLQFICTQKKP--DQKDI<br>QFQFQLEVTVLDTDYATYAVMYRCVQFPQEL-GSMFEDNTLLLQRDVNKGVDEN-KIESK<br>LN---LSFDSFRSRNDVVGGCRKLPSK-KEKYIVINLVL
>E2J710_triafestinlike	--------------DYPP-IAKCNHPSAMANFNQKKFLSGKWYVTKAEHGSDSTVCRQYK<br>GKFNNEKQQFIGDGYYTFQ-NQTFYFTVRCSRVQ--NTDGQPPMKFICTQKNP--DIAQM<br>TFQFQLEVTVRDTDYRTYAVMYRCVQFPQEL-GSHFEDNTLLIQRNANLEVDEN-KIEKT<br>LS---LSFDSFRSRNDVNGGCPELPSK-KKNKKPKT---
>A6YPF2_triafeslike	--------------DYPS-IENCTHPPAMANFNPKKFLEGKWYVTKAKHGSNSTVCREYR<br>AKTKDKDQILVGDGYYSFN-GGTFYFTVRCKRLP--NNEVQKPLQFTCTQKSP--DVASM<br>QFQFQLEVTILDTDYKNYAVMYRCVQFPEKL-GAHFEDNTLLLHRNPEQLVDEN-LIESK<br>LR---MSFDSFRSREDVVDGCPKLPSK-KKNKAS-----

>A7BJ46_triafestin-2	--------------DYPS-IENCTHPPAMANFNQKKFLEGKWYVTKAKHGSNSTVCREYR<br>AKTKGNDQILVGDGYYSFN-GGTFYFTVRCKRLP--NKEVQKPLQFTCTQKST--DDPSK<br>MFKFQLEVTILDTDYANYAVMYRCVQFPEQL-GSHFEDNTLLLHRNPDQLVDEN-QVERK<br>LN---LSFDSFRSREDVVDGCPKLPSK-KKNKAS-----
>A7BJ45_riafestin-1	--------------DYPS-IPKCTHPPAMANFNQKKFLEGKWYVTKAKHGSNSTVCREYR<br>AKTKGNDQILVGDGYYSFN-GGTFYFTVRCKRLP--NKEVQKPLQFTCTQKSP--DDPSK<br>MFKFQLEVTILDTDYANYAVMYRCVQFPEEL-GSHFEDNTLLLHRKLDQLVDEN-LIERK<br>LK---LSLPSFKSRDDVVEGCRELPSK-KKKTKP-----
>A6YPF6_triafes2like	--------------DYPP-IEKCNHPPAMTKLNQKKFLNGTWYVTKAKHGSDSTVCRQYK<br>AKFNDKKQQFIGDGYYTFQ-NQTAYFTVRCTRQPRNNKNTKKRMQFICTQKNP--DDERM<br>QFRFQLEVTVLGTDYANYAVMYRCVQFPPEL-GSQFEDNTLLLHRNPEQLVDEN-QVERK<br>LN---LSFDSFRSREDVVDGCPNLPSK-KKNKAS-----
>A6YPF4_triafetinlike	--------------DYPP-IEKCNHPPAMTNLNQKKFLNGTWYVTKAEHGSDSTVCRQYK<br>AKFNDKKQQFIGDGYYTFQ-NQTAYFTVRCSRQPRNNKNTKKRMQFICTQKNP--AVESM<br>IITFQLEVTVLDTDYANYAVMYRCVQFPKQL-GSHFEDNTLLLHRNPDQLADEN-LIESK<br>LS---MSFDSFRSREDVVDGCPKLPSK-KKNKAS-----
>A6YPR8_triafestinlike	--------------DYPTGLTECKHPIAMANFNPKKFLHGKWFVTNAKHGSNSTVCREYK<br>AKQNGRNQELIGDGYYSFS-GQTIYFTVRCKRLPNKKQQKQQPLKFTCTQKNP--NVKSM<br>KILFQLEVTVLHTDYANYAIMYRCVKFPPEL-QSLIEDNTLVLQRKANKPVDEDSCIHKI<br>LKQQDLLLESFKSRKGVK--CLKPPKK-KKN--------
>A6YPR9_triafeslike	--------------DYPTGLTECKHPIAMANFNPKKFLDGKWFVTNAKHGSNSTVCREYK<br>AKSKGNNQELIGDGYYSFS-DQTVYFTVRCKRLPKEKKQKQQPLKFTCTQKNP--TDKKL<br>KIPFQLEVTVLHTDYADYAIMYRCVKFPPEL-KSLIEDNTLVLQRKANKPVEKDSCVDKI<br>LKKQGLSLESFKSRKGVV--CTPPPKK-KKV--------
>E2J709_triafestinlike	--------------QYEK-IPNCNPPEATKNLDTESFLKGKWYVTNAKQGSNSTVCREYR<br>TKNKDGKQVLVGDGYYTFN-GQKPYFKVRCKRQS----PT--ELSYTCTQTMP--GNKEL<br>KNQFQLQLTILHTDYTNSAVMYRCVQFPPEL-GSHFEDNILVLHRDPSITNDNDNAVRNA<br>LKSQGLQLNSLKSREGVV--CPEPPPK-IKE--------
>E2J753_triafeslike	--------------EYAQ-IPNCTPPEAMANLDTARFLEGKWYVTNAKQGSNSTVCREYR<br>TKTN--KEVLVGDGYYTFM-NQRLYFKVRCKKQS----QT--ELSYTCKQTMP--DNSEM<br>KNQFQLQLTILHTDYTNSAVMYRCVQFPPEL-GSNFEDNILVLHRDPSITNDNDNAVRNA<br>LKSQGLQLNSLKSREGVV--CPEPPPKRIKI--------

>E2J717_triafeslike	--------------EYAQ-IPNCTPPEAMANLDTARFLEGKWYVTNAKQGSNSTVCREYR<br>TKTN--KEVLVGDGYYTFM-NQKLYFKVRCKKQS----QT--ELSYTCKQTMP--DNSEM<br>KNQFQLQLTILHTDYTNSAVMYRCVQFPPEL-GSNFEDNILVLHRDPSITNDNDNAVRNA<br>LKSQGLQLNSLKSREGVV--CPEPPPKRIKI--------
>D1MWC9	--------------DYPK-LDNCNPPEAMKGLDSGKFLKGTWYVTNAQCGSNSTVCREYK<br>GKRENGNSVLNGNGYYSFG-SQKVYFEVRCNKQS----NSNYKLTFDCTQKGP--AGSGK<br>NFQLQLEVTVLYTDYSDLAIMYRCVKFPPQL-GSLIEGNVLVLRRDASKTNDKNPKIEET<br>LKKQGWSLDTFNSREGVT--CPEPPQK------------
>D1MWD0	--------------DYPK-LEKCNPPEAMEGLDSGKFLKGTWYVTNAQYGSNSTVCREYK<br>GKRENGNPVLNGDGYYSFG-SQKFYFEVSCKKQS----NRNYKLTFGCTQNGP--KDSGM<br>NFQLQLEVTVLYTDYSDLAIMYRCVKFPREL-GSLIEGNVLVLRRDASKTNDKNPKIEET<br>LKKQGWSLNTFNSREGVT--CPEPPQK------------
>27479c2s12	---WYVTNAKHGSNSTVCREYN<br>GKRENGNPVLNGDGYYSHG-NLKIYFEVRCDKQS----DTNYKLTFSCTQKGP--AGTNM<br>NFQFQLEVTVLSTDYDDFAIMYRCVKFPPQL-GSRIEDNVLVLHRKDCKTEDKD-RVEET<br>LKKNGWSLDSFKSRKGVN--CPEPPQK------------
>27479c2s8.5	---WYVTNAKHGSNSTVCREYN<br>GKRENGNPVLNGDGYYSFR-NLKIYFEVRCDKQS----DTNYKLTFSCTQKGP--AGTNM<br>NFQFQLEVTVLSTDYDDFAIMYRCVKFPPQL-GSRIEDNVLVLHRDATKTNDKNPQIEDI<br>LKKQDWSLDTFNSRDGVE--CPLPPQK------------
>27479c2s9.5	---WYVTNAKHGSNSTVCREYK<br>GKRENGNPVLNGDGYYSVG-SQKIYFEVRCDKQS----DKNYKLTFSCTQKGP--AGSKM<br>NFQFQLEVTVLSTDYDDFAIMYRCVKFPPQL-GSRIEDNVLVLRRNPNTDNKNP-LIETT<br>LKSQDLSLDTFKSRKGVT--CPKLPQK------------
>27479c2s11.5	---WYVTNAKHGSDRTVCREYK<br>GKRQNGIRQLIADGYYSYGQDQTAYFNVLCHKQS----DRNYKLTFNCTQRGSITDDQKV<br>IIQFQLEITVLLTDYDDFAIMYRCVKFPPQL-GSRIEDNVLVLRRNPNTDNKNP-LIETT<br>LKSQDLSLDTFKSRKGVT--CPKLPQK------------
>27479c2s14	---WYVTNAKHGSDRTVCREYK<br>GKRQNGIRQLIADGYYSYGQDQTAYFNVLCHKQS----DRNYKLTFNCTQRGSITDDQKV<br>IIQFQLEITVLLTDYDDFAILYRCGKIPSSS-GSRIEDNVLVLHRKDCKTEDKD-RVEET<br>LKKNGWSLDSFKSRKGVN--CPEPPQK------------

>G8JKH9_triafestinlike	RGSKFFGIVTFAFADYTK-IQNCEQPEAMQNFDANKFLKGTWYVTNAKQGSESTVCREYK<br>AGME--MTNLTETDTTAFK-VKKTYFQVSCKKKS----ENKGKLTFQCTQRGTV-SGQMM<br>SIQFDLDVTIVSTDYNSYAVMYRCAKFPKEL-QSRIEDNVLILRRDAKQTEIEQ-SIKTT<br>LQNQGWPSDKFISRKHGT--CPTPPQK------------
>G8JKI0_Triafestin2Like	--<br>----------GGDGYYSFQ-GQKTYFQVSCKKKS----ENNGKLTFQCTQSGTV-SGNEM<br>NFQFQLEVTIVSTDYNNYAVTYRCVKLPTEL-GGGYEDNVLILRRNAKQTEIEQ-SIKTT<br>LQNQRWPSDKFISRKDGT--CQKPPQK------------
>G8JKI1	--<br>----------------------------RGKKKS----ESNGKLTFQCTQSGTI-SGKTM<br>SIQFDLDVTIVSTDYNSYAVMYRCVKFPEEL-GSRIEDNVLILRRDAKQTEVE--SIKAT<br>VKNQEWTLDKFISRKDDT--CSKLSQK------------
>G8JKD7_Triafestinlike	--------------DYTK-IQNCEQPEAMQNFDANKFLKGTWYVTNAKQGSESTVCREYK<br>AGNE--NGELNGDGYYNFK-GQKTYFQVSCKKTS----ENKGKMTFQCTQRGTV-SGNEM<br>NFQFQLEVTIVSTDYNSYAVMYRCVKLPTEL-GGGYEDNVLILRRDAKQTEVE--SIKAT<br>VKNQEWTLDKFISRKDDT--CSKLSQK------------
>G8JKI2_triafestinLike	--TAGATDLTVCREYK<br>ASQN-ENGELIGDGYYNFN-GQKTYFEVRCKKKS----ESNGKLTFQCTQSGTI-SGQKM<br>SIQFDLDVTIVSTDYNNYAIMYRCVKLPTER-GGGYEDNVLVLHRDSSKTDDNV-NVEQT<br>LKSKGWQFSSFISRKGRN--CQ-----------------
>G3CJN2_triafestinlike	--<br>----------INNNSLSRP-GQPSYFQVRCKDPS----NNNGKLTFSCKQSGTV-GSKKM<br>DIPFQLEVTILSTDYNDFAVMYRCAKLPSSTGGTHFEDNVLVLHRVSTKTEDKR-NVGET<br>LKKQGWSLDDFNSRKGVD--CPAPP--------------
	Triatin subfamily
>E2J756	SEEIQQPKQKLVYGQDKCQGYGGMKDLDTEKFFKGNWSLIYSTPSPRLSHSNMCRDYKIT<br>PYKNDTVEVTYEYDENRCGNHYVAKCYGNQNSTRIDQFDFDCYLHNEREELTSTHVNAYF<br>LATDYDSYSVAYRCVQNEDHF-EDNVFILFRPGKTDEDYTKTIAEHYGLTMGDFLARKDA<br>TC---
>E2J758_triatinlike	SEEIQQPKQKLVYGQDKCQGYGGMKDLDTEKFFKGNWSLIYSTPSPRLSHSNMCRDYKIT<br>PYKNDTVEVTYEYDENRCGNHYVAKCYGNQNSTRIDQFDFDCYLHNEREELTSTHVNAYF<br>LATDYDSYSVAYRCVQNEDHF-E---------GKTDEDYAKTIAEHYGLTMGDFLARKDA<br>TC---

>E2J757_triatinlike	SEEIQQPKQKLVYGQDKCQGYGGMKDLDTEKFFQGNWSLIYSTRSPRLSHSNMCRDYKIT PFGNGTVEVTYEYDENRCGNHYVAKCYGNQNSTRIDQFDFDCYLHNEREELTSTHVNAYF LATDYDSYSVAYRCVQNEDHF-EDNVFILFRPGKTDEDYAKTIAEHYGLTMSDFLARKDA TC---
>A6YPQ0_triatinlike	SEEITQTKQKLVYGQNKCQGYGGMINLDPKRFFKGNWSLIYSTPSSRLSHSNMCRDYKIT PYENRTVEVTYEYDENRCGNHYVAKCYGNQNSTREDQFDFDCYLHNEREELTSTHVNAYF LATDYDNFSVAYRCVQSKDHF-EDNIFILFRPDKTDEDYANTIAEHYGLTMDDFLARKDA PC---
>A6YPQ1_triatinlike	SEEITQTKQKLVYGQNKCQGYGGMINLDPKRFFKGNWSLIYSTPSSRLSHSNMCRDYKIT PYQNGTVEVTYEYDENRCGNHYVAKCYGNQNSTREDQFDFDCYLHNEREELTSTHVNAYF LATDYDNFSVAYRCVQSKDHF-EDNIFILFRPDKTNEDYAKTIAEHYGLTMCDFLARKDA TC---
>E2J720_triatin	----SEQKQKLEYGQGSCKRYGGIADFDPKKFFSGNWSLTHATRSTRVTDSTVCRDYDLK MHDNGTIEAMYGYNENRCGNPYDVHCYGTQNSSRKDEFNFDCHLHNDREEKMDTHIDAYF LATDYDTYCVVYRCVTTTEYF-EDNLFILFRPGKTDESYAKILVENYGLTMNDILARKDA TCAKS
>E2J7I1_triatinLike	----------------------------KKFFSGNWSLTHATRSTRVTDSTVCRDYDLK MHDNGTIEAMYGYNENRCGNPYDVHCYGTQNSSRKDEFNFDCHLHNDREEKMDTHIDAYF LATDYDTFCVVYRCVTTKEYF-EDNLFILFRPNKTDEDYAKVLVENYGLTMDDILARKDA TCAKS
>A6YPP0_triatinlike	----SEEKQRIQYGQNNCQGYRSMQNFTQTQFFQGIWSLTHSTPSTRVTASTICRDYEVT VHQNGTIQVTYGYNENECGNHYDVHCNGTENSDTKGVFNFDCHLYNGMEESQNTHIDVAF LATDYETYCVVYRCVKIDENFVEDNVFILYRPDKTNEDYAKTLAEHYGLSIDDFISRKNA SCTTR
>A6YPN9_triatinlike	----SEEKQKIQYGQSNCQGYRSMQNFTQTQFFQGKWSLTHSTPSTRVTASTICRDYEVT VHQNGTIQVTYGYNENECGNHYDVHCNGTENSDTKGVFNFDCHLYNGMEESQNTHIDVAF LATDYDNYCVVYRCVKIDENFVEDNVFILYRPGKTNEDYAKTLAEHYGLTIDNFISRKNA SCTSR
>E2J768_triatinlike	----SVEKPKIQYKQGYCQGYRSFNDFDPKRFFKGNWSLTHSTPSNRVTASTICRDYELK EHENGTIQATYGYNENECGNRYDVHCNGTENSKLLGEFNFDCHLYNGEEES-KTHIDAAF IATDYEKYCVVYRCVTIDGQFAEDNIFILFRPDKTDEELAKKIAEHYGLTIDDFISRKNA TCTNK

	Dipetalodipin and pallidipin
>G3CJP9_pallidipin2	RAINAAILLGILMHAFSEECTLKSPAENFDADKYFDVPHVYVTHSKSGPKEKVCREYNTT<br>KNSDKTTSTTVVTL--KTGGT-QSIILSCTNTPKTGKNGQYSMDCKAPNSKDG--KNIQL<br>ESSIIATDNQNYALIHFCPETPSGTITEDILVVQTNKDGVNEGVTSAIKNYGWSIENWIS<br>RKDAGCQ----
>G3CJM8_PallidipinLike	--NGPKEKVCREYNTT<br>KNSDKTTSTTVVTL--KTGGT-QSIILSCTNTPKTGKNGQYSMDCKAPNSKDG--KNIQL<br>ESSIIATDNQNYALIHFCPETPSGTITEDILVVQTNKDGVNEGVTSAIKNYGWSIENWIS<br>RKDAGCQ----
>G3CJP8_pallidipin2	---KEKVCREYNTT<br>RNSNKTTSTTVVTL--KTGGT-QSIILSCTNTPKTGKNGRYSMDCKAPNSKDG--KNIQL<br>ESSIIATDNQNYALIHFCPETPSGTITEDILVVQTNKDGVNEGVTSAIKNYGWSIENWIS<br>RKDAGCQ----
>G3CJM9_pallidipin2	--ERVCREYNTT<br>KNSGGPTSTTKVIS--KTGGT-QSIILSCTNTPKTGKNGQYSMDCKAPNSKDG--KNIQL<br>ESSIIATDNQNYALIHFCPETPSGTITEDILVVQTNKDGVNEGVTSAIKNYGWSIENWIS<br>RKDAGCQ----
>G3CJN5	---GQRKKXCREYNTT<br>KNSDKTTSTTKVTS--KTGGT-QSIVLSCNNSPKSGVKGQYFMDCQVPGGTGG--NNIQL<br>ESSIIATDNKNYALVHFCPITGRGVVTEDIVVLQTNKDNVDPGVTSAFRTNGWSLQEWTS<br>RKDVTCK----
>G3CJS0_dipetalodipin	-----------------KECTLMAAASNFNSDKYFDVPHVYVTHSKNGPKEKVCREYNTT<br>KNSDKTTSTTVVTL--KTGGT-QSIVLSCNNSPKSGVKGQYFMDCQVPGGTGG--INIQL<br>ESSIIATDNKNYALVHFCPITGRG-VTEDIVVLQTNKDNVDPGVTSAIKNYGWSLENWKS<br>RKDAGCQ----
>G3CJP1_pallidipin2	---HSRNGTNEEVCREYNTT<br>KNSDKTTSTTVVTL--KTGG--QGQKLSCTNTPKTGSAGQFSVECQGQDG-----TNIQL<br>ETSVIATDYQKYALLQTCLKSGSGAITDDILVLQTKKDGVDPGVTSVFTTNRWSIETWHS<br>RAKANCD----
>D1MWE1_pallidpin2like	-----------------KECELMPPAENFDSDKYFNIPHVYVTYSKNGPQEKVCREYKTT<br>KNSDGTTNTEVIV---KTGGQQQKTVLTCTNKEKNGKPGQYSVECEVPNG----NHKIQL<br>ETSVFATDNKNYALLQSCNKDGKS--DYDIFVLQTNKDVVDPGVTSAFNLAKWSLKDWVS<br>RSNVDCNNIQN

>D1MWE0_pallidipinLike	------------------ECELMPPASNFDSKKYFNIPHVYVTHSKNGPDKKVCREYKTT<br>KNSNGTTNTEVTV---KTGGK-EQTLLKCINKEKNGKAGQYSVECEVPNG----NHKIQL<br>ETSVFATDNKNYALLQSCNKNGKN--DFDIFVLQTNKDG-DPGVTAVFDLTKWPLDKWVS<br>RSNVNC-----
>27479c2s4_pallidipinlike	-----------------KECELMPPASNFDSEKYFDIPHVYVTHSRNGPKENVCREYKTT<br>KSTGGLTLTDVIV---NTGGK-QRTVLNCINTVKNIKPGHYFLECEAPNGD---NQKIQL<br>E---<br>-----------
>27479c2s13_pallidipinlike	-----------------KECELMPPASNFDSEKYFDIPHVYVTHSRNGPKENVCREYKTT<br>KNTDGTTHTEVIV---KTGGK-QRTVLNCINTQKNGKPGQYSVECEVPNGNGG-NQKIQL<br>ETSVFATDNEKYALLQSCNKDGES--EDDIFVLQTNKDPVEQGVTSVLKSVNWSLNDWFS<br>RSKVNCDNMK-
>27479c2s3_pallidipinlike	-----------------EECELTPPEDNFDLEKYFSIPHVYVTHSRNGPKEQVCREYKTT<br>KNSDGTTTTTLVTSDYKTGGKPYHSQLKCTNTPKNGGKGQFSVECEVPNGNGG-KKKIHV<br>ETSVIATDYKNYALLQSCTKTESG-IADDVLLLQTKKEGVDPGVTSVLKSVNWSLDKWIS<br>RTEVNCDNIQN
>Q27042_pallidipin	-----------------EECELMPPGDNFDLEKYFSIPHVYVTHSRNGPKEQVCREYKTT<br>KNSDG-TTTTLVTSDYKTGGKPYHSELKCTNTPKSGVKGQFSVECEVPNGNGG-KKKIHV<br>ETSVIATDYKNYALLQSCTKTESG-IADDVLLLQTKKEGVDPGVTSVLKSVNWSLDDWFS<br>RSKVNCDNMK-
>27479c2s7_pallidipinlike	-----------------KECELMPPASNFDSEKYFDIPHVYVTHSRNGPKEQVCREYNTT<br>KIQGDTTPYTVVTSDYKIRGETHHSQLKCTNTPKNGGKGQFSVECEISNGNGGNKKKVQF<br>ETSVFATDYKNYALLQSCTKTESG-IADDVLLLQTKKEGVDPGVTSVLKSVNWSLDDWFS<br>RSKVNCDNMK-
>27479c2s6_pallidipinlike	-----------------KECQLMPATDDYNADQYFSIPRVYAIYSKNGPEENVCREYETK<br>KKTDGTIVTTVSGD-KKNRGQ-QQTVLACTNREKSGSKGQFFVECEVPGKNGG-NMKIQV<br>ETSVLATDN---<br>-----------
>G8JKI8_pallidipin2Like	-----------------------P-----------GVPQVYMTLSKNGPKEKLCREYKTT<br>ESS--TTVNNLVIL--EKRGN-EQTQLNCIETLKSDRKGQFSVECELPGNSG---KKVQF<br>ESSVIATDNKNYAILQTCPKTESSIVTEDIVVLQTNEEGVEEGVTNYFTSQGWSLDTWHS<br>RKKVKC-----

	Triplatin and infestilin subfamily
>A6YPE4_triplatinlike	----ETCTLIDAA-PNFDADKYFKISHAYATYSQNREPETTVCREYSTT-TRDGKIITTF<br>TID--DRTLRT--NVECTNTPI-TGKNGQFTSNCRLSAGNTITVTSSILATDNKSYAILQ<br>RC-------SQNGPGNILVLQTNKDGGVPQGVTDYFKGKGWDINKWISRKEANCR----
>Q6UN99_triplatinlike	----ETCTLIDAA-PNFDADKYFKISHAYATHSQNREPETTVCREYSTT-TRGGKIITTF<br>TIN--DRTLRT--DVECTNTPI-TGKKGQFTSNCRLSAGNTITVTSSILATDNKSYAILQ<br>RC-------SQNGPGNILVLQTNKDGGVPKGVTDYFKGKGWDINKWISRKEANCR----
>Q18NS6_triplatin-2	EVNSETCTLMEAA-KNFDENKYFNIPLAYATHSKNREPETNVCREYSTARGPDGKTVTTF<br>TIK--DKTLTS--AVKCTNTPI-PGSNGQFSSDCELSAGNRITVTTSILATDNEKYAILQ<br>RC-------PTSGPGNILVLQTNKNGVEQ-GVQNYFNQKGWDISTWLSRTTVGC-----
>A6YPH4_triplatinlike	----EECVLKPGA-SNFDSKKYFDIPLAYVTHSQN-DPETNVCRDYQSSRRADGKPVTSF<br>TIT--DRSYPTGAKVTCVNNEI-KGEKGHFTSVCELPAGNKYEVKSSILATDNESYAVLQ<br>RC-------GTSGPGNILVLQTKKNGVNP-AVTKYLNANGWDITKWISRQTVRC-----
>Q45KX1_infestilin	EVNSETCTLMEAA-KNFDENKYFSIPLAYATHSQNREPETTVCRDYRTT-KTDGKIVTTF<br>TIN--DKIAPKDTQVSCINTPT---SKGQFSSECTLPAGTKIKVTTSILATDNANYAVFR<br>D----------------ALRVDQ-----------------VIFWYYRKIKMAP----
>Q18NS7Triplatin-1	----EECRLMQPA-ANFDAATYFSIPHVYVTHSKN-EPKTDVCREYDTSKT-DGGSTTVI<br>TSNYKIKGQAVNNKVTCTSTGLKNGQTGQFSVVCQPPTGAAVTLTTSVLATDNQNYAILQ<br>RC-------PTSGQGNILVLQTAKEGVNP-GVKDFFQKKGWNIDSWFSRTNVNCENIQS
>A6YPS0_pallidipinlike	----QECQLMQPA-ANFDAPTYFSIPHVYVTHSKN-EPKTDVCREYDTSKT-DGGSTTVI<br>TSYYKIKGQAVNNKVTCTSTGLKNGQTGQFSVVCQPPTGTAVTLTTSVLATDNQNYAILQ<br>RC-------PTSGQGNILVLQTTKEGVNT-RVTDFFKKQSWNIHSWISRTKVNCENIES
>Q6UNA0_pallidipinlike	----EECRLMQPA-ANFDAATYFS-PHVWVTHSKN-EPKTDVCREYDTSKNGDGSTTTVI<br>TSNYISRGVAVNNKVTCTSTGL-SGQKGQFSVVCQPPTGTAITLTTSVLATDNQNYAILQ<br>RC-------PKSGQGNILVLQTTESGVNP-GVKRYFDDNGWSIGSWFSRTNVNCKNIEN
>E2J754_pallidipinlike	----ENCDLKQPV-ANFDSEKYFSISRSFVTYSKN--DQKPVCREYLTTRNTDGTTKTVY<br>TIR--DRTAPSDVEVNCINKPK-SGSNGQFSVSCTLPAGNTFQLTTSVVDTDYNKYVILQ<br>SC-------PDSGPGDILVFQTDKNEKIP-AVTNYIAQQG---GQWYSRINDRC-----
>Q0MTD7_pallidipin2like	----EDCVLKPTCRQTFNSEQYFAIRHSYVTYSKN-GPETKVCREYVTTKNTNGTTTTVY<br>TIK--DQTNPSGSNATCINTPK-TGSNGQFSVTCTLSAGNEFQLTTSVVDTDHKTYVILQ<br>VCLNSGPGITDSGPGDILVLQTDKNVEIP-AVTKYIQGNT---GQWYSRKNSRC-----

	Dimiconin and triabin subfamily
>E2J756	SEEIQQPKQKLVYGQDKCQGYGGMKDLDTEKFFKGNWSLIYSTPSPRLSHSNMCRDYKIT<br>PYKNDTVEVTYEYDENRCGNHYVAKCYGNQNSTRIDQFDFDCYLHNEREELTSTHVNAYF<br>LATDYDSYSVAYRCVQNEDHF-EDNVFILFRPGKTDEDYTKTIAEHYGLTMGDFLARKDA<br>TC---
>E2J758_triatinlike	SEEIQQPKQKLVYGQDKCQGYGGMKDLDTEKFFKGNWSLIYSTPSPRLSHSNMCRDYKIT<br>PYKNDTVEVTYEYDENRCGNHYVAKCYGNQNSTRIDQFDFDCYLHNEREELTSTHVNAYF<br>LATDYDSYSVAYRCVQNEDHF-E---------GKTDEDYAKTIAEHYGLTMGDFLARKDA<br>TC---
>E2J757_triatinlike	SEEIQQPKQKLVYGQDKCQGYGGMKDLDTEKFFQGNWSLIYSTRSPRLSHSNMCRDYKIT<br>PFGNGTVEVTYEYDENRCGNHYVAKCYGNQNSTRIDQFDFDCYLHNEREELTSTHVNAYF<br>LATDYDSYSVAYRCVQNEDHF-EDNVFILFRPGKTDEDYAKTIAEHYGLTMSDFLARKDA<br>TC---
>A6YPQ0_triatinlike	SEEITQTKQKLVYGQNKCQGYGGMINLDPKRFFKGNWSLIYSTPSSRLSHSNMCRDYKIT<br>PYENRTVEVTYEYDENRCGNHYVAKCYGNQNSTREDQFDFDCYLHNEREELTSTHVNAYF<br>LATDYDNFSVAYRCVQSKDHF-EDNIFILFRPDKTDEDYANTIAEHYGLTMDDFLARKDA<br>PC---
>A6YPQ1_triatinlike	SEEITQTKQKLVYGQNKCQGYGGMINLDPKRFFKGNWSLIYSTPSSRLSHSNMCRDYKIT<br>PYQNGTVEVTYEYDENRCGNHYVAKCYGNQNSTREDQFDFDCYLHNEREELTSTHVNAYF<br>LATDYDNFSVAYRCVQSKDHF-EDNIFILFRPDKTNEDYAKTIAEHYGLTMCDFLARKDA<br>TC---
>E2J720_triatin	----SEQKQKLEYGQGSCKRYGGIADFDPKKFFSGNWSLTHATRSTRVTDSTVCRDYDLK<br>MHDNGTIEAMYGYNENRCGNPYDVHCYGTQNSSRKDEFNFDCHLHNDREEKMDTHIDAYF<br>LATDYDTYCVVYRCVTTTEYF-EDNLFILFRPGKTDESYAKILVENYGLTMNDILARKDA<br>TCAKS
>E2J7I1_TriatinLike	-----------------------------KKFFSGNWSLTHATRSTRVTDSTVCRDYDLK<br>MHDNGTIEAMYGYNENRCGNPYDVHCYGTQNSSRKDEFNFDCHLHNDREEKMDTHIDAYF<br>LATDYDTFCVVYRCVTTKEYF-EDNLFILFRPNKTDEDYAKVLVENYGLTMDDILARKDA<br>TCAKS
>A6YPP0_triatinlike	----SEEKQRIQYGQNNCQGYRSMQNFTQTQFFQGIWSLTHSTPSTRVTASTICRDYEVT<br>VHQNGTIQVTYGYNENECGNHYDVHCNGTENSDTKGVFNFDCHLYNGMEESQNTHIDVAF<br>LATDYETYCVVYRCVKIDENFVEDNVFILYRPDKTNEDYAKTLAEHYGLSIDDFISRKNA<br>SCTTR

>A6YPN9_triatinlike	----SEEKQKIQYGQSNCQGYRSMQNFTQTQFFQGKWSLTHSTPSTRVTASTICRDYEVT VHQNGTIQVTYGYNENECGNHYDVHCNGTENSDTKGVFNFDCHLYNGMEESQNTHIDVAF LATDYDNYCVVYRCVKIDENFVEDNVFILYRPGKTNEDYAKTLAEHYGLTIDNFISRKNA SCTSR
>E2J768_triatinlike	----SVEKPKIQYKQGYCQGYRSFNDFDPKRFFKGNWSLTHSTPSNRVTASTICRDYELK EHENGTIQATYGYNENECGNRYDVHCNGTENSKLLGEFNFDCHLYNGEEES-KTHIDAAF IATDYEKYCVVYRCVTIDGQFAEDNIFILFRPDKTDEELAKKIAEHYGLTIDDFISRKNA TCTNK
	Procalin subfamily
>G3CJS2	V-TGSECNMPSPMAGFEKSKFFTGMWYVTHETNVTTPSECNTLTTRLENNKVFVEHKYVK DGKTGTLVCEGQEDGQNMFPLNCKFNGVTMEEVTRIVMDTDYNDYALYYLCTAYKSGVNT GKKAEHYIISRREPKDDIPEKLRSQAATLQLQKCGKVAS
>G3CJR6_Procalinlike	QNVQNGCKMPTPMANLDATKFFQGTWHVTHETNVTIPSECNTLTTSKKGDKVVVEHKYTK DGKEGRLICEGQEDGQNMFPLNCKFEGETLEEVTRIVMNTDYNDYALYYLCTTYKSGVNA GKKAEHYIISRRDPNKEIPDHLQSKVKELNLQKCGQNTG
>Q9U6R6_procalinTriatoma protracta	----DECENPEPMQGFSASQFYQGXWYVTHETSAXTLSECNILTTSNDNGKFTVKHKYTK DGXVGELICEGQASANNKFTYDCKFXGZTMEQVTRTAMDTDYNDYALYYLCTTYKXGPNA GKKEGHYILSRRQPNTEIPDALKTKTKDLNLKLCG----
	**Proteins without biological function**
	T. matogrossensis
>E2J750	--------ATTGISECKTVTPMDRFSATRFFQRTWYVSHVQKKTSNTVCQTFKTSSPSDG VYAIEYTF----GDNNVRCEATREKEKKLTFTCKNGDTTIFTALFVIMDTDYENYALFYR CVTM-TGGYKDDNYLVLSSTSGDQQIPTSLTSLTSSLNLLWCKDIRASVV
>E2J746	--------ATTGISECKTVTPMDRFSATRFFQRTWYVSHVQKKTSNTVCQTFKTSSPSDG VYVIEYTF----GDNNVRCEATREKEKKLTFTCKNGDTTIFTALFVIMDTDYENYALFYR CVTM-TGGYKDDNYLVLSSTSGDQQIPTSLTSLTSSLNLLWCKDIRASVV
>E2J751	--------ATTGISECKTVTPMDRFSATRFFQRTWYASHVQKKTSNTVCQTFKTSSPSDG VYVIEYTF----GDNNVRCEATREKEKKLTFTCKNGDTTIFTALFVIMDTDYENYALFYR CVTM-TGGYKDDNYLVLSSTSGDQQIPTSLTSLTSSLNLLWCKDIRASVV
>E2J752	--------ATTGISECQSVTPMENFSATKFFQRTWYVSHVQKKTSNTVCQTFKTSSPSDG VYVIEYTF----GDNNVRCEATREKEKKLTFTCKNGDTTIFTALFVIMDTDYENYALFYR CVTM-TGGYKDDNYLVLSSTSGDQQIPTSLTSLTSSLNLLWCKDIRASVV

>E2J7H9	GILTYAYGATSGISECKTATPMDRFTATRFFPRTWYVSHVQKKTSNTVCQTFKTSSPSDG<br>VYVIEYTF----GDNNVRCETTREKEKKLTFTCKNGDTTIFTALFVIMDTDYENYALFYR<br>CVTM-TGGYKDDNYLVLSSTSGDQQIPTSLTSLTSSLNLLWCKDIRASVV
>E2J749	--------ATTGISECKTVTPMDRFSATRFFQRTWYVSHVQKKTSNTVCQTFKTSSPSDG<br>VYVIEYTF----GDNNVRCEATREKEKKLTFTCKNGDTTIFTALFVIMDTDYENYALFYR<br>CVTM-TGGYKDDNYLVLSSTSGDQAIPASLTSLTSSLGLKSCEEIKTMVY
>E2J747	--------ATTGISECKTVTPMDGFSATRFFTRTWYVTHVQKKTSNTVCQTFTASKPSNT<br>TYAVDYTFTGDNGENNVHCVATRTEEKKLTFNCKNGDTPIFDAVFVIMATDYNNYALFYR<br>CVTMKSSRVKDDNFLVLSSTSGDQAIPASLTSLTSSLGLKSCEEIKTMVY
>E2J716	--------ATTGISECKTVTPMDGFSATRFFTRTWYVTHVQKKTSNTVCQTFTASKPSNT<br>TYAVDYTFTGDNGENNVHCVATRTEEKKLTFNCKNGDTPIFDAVFVIMATDYNNYALFYR<br>CVTMKSSGVKDDNFLVLSSTSGDQAIPASLTSLTSSLGLKSCEEIKTMVY
>E2J748	--------ATTVISECKTVTPMDGFSATRFFTRTWYATHVQKKTSNTVCQTFTASKPSNT<br>TYAVDYTFTGDNGENNVHCVATRTEEKKLTFNCKNGDTPIFDAVFVIMATDYNNYALFYR<br>CVTMKSSGVKDDNFLVLSSTSGDQAIPASLTSLTSSLGLKSCEEIKTMVY
>E2J711	--------ATTGISECKTVTPMDRFSATRFFTGTWYVTHVQKQTSQTVCQTFTASKPSNT<br>TYAVDYTFTGDNGENNVHCEATRTEEKKLTFNCKNGDTPIFTAVFVVMATDYTDYALFYR<br>CVTM-TSGYKDDNYLVLSRTSGNQEIPTSLTSLTSDLNLLSCADISTPVV
>E2J760	--------DSSGISQCKTVEAKTDFRATNFFTGTWYVTHVQKKTSNTVCQTFTASKISNT<br>TYVVEYTFTDNNGQNNIRCEAERGEHQQMTFTCKNGGNTIFTAVFVVMATDYTDYALFYR<br>CVTMTSTGNKDDNYLVLSRTNGDQAIPASLTDLTSSLNLRSCADLRTRVV
>E2J726	--------TSSGISECKTVDAKTDFSGTQFFTGTWYVTHVQKKTSNTVCQTFAASKPSNT<br>TYVVDYTFTNDNGKNNIRCEAERGEHQKMTFICKNGDTPIFTAIFVVMATDYTDYALFYR<br>CVTMLSTGNKDDNYLVLSRKSGNEALPTSLTSLTSDLNLLSCQTI-----
	T. rubida
>G8JKC7	E---MVTVAECGGAQVNAMDNYRAPDYHKEKWYVTHVTKVTEPTECRTLTATTKSDGKTF<br>TVEHPFGDGGSQTLHCEAQAEAAKRLTFTCKVGDQVVDKTIFITVDTDYNDYSLYYLCIA<br>PTGGTPHDTYLIARRKAG-DNIPPQLESFTQGMDFKK--------C------
>G8JKC9	E---MVTVAECGGAQVNAMDNYRAPDYHKEKWYVTHVTKVTEPTECRTLTATTKSDGKTF<br>TVEHPFGDGGSQTLHCEAQAEAAKRLTFTCKVGDQVVDKTIFITVDTDYNDYSLYYLCIA<br>PTGGTPHDTYLIARRKAG-DNIPPQLESFTQGMEFKK--------C------

>G8JKD2	E---MVTVAECGGAQVNAMDNYRAPDYHKEKWYVTHVTKVTEPTECRTLTATTKSDGKTF TVEHPFGDGGSQTLHCEAQAEAAKRLTFTCKVGDQVVDKTIFITVDTDYNDYSLYYLCIA PTGGTPHDTYLIARRKAG-DNIPPQLESFTQGMDFKKMLVILQNECPKTFVV
>G8JKE5	E---MVTVAECGGAQVNAMDNYRAPDYHKEKWYVTHVTKVTEPTECRTLTATTKSDGKTF TVEHPFGEGGSQTLHCEAQAEAAKRLTFTCKVGDQVVDKTIFITVNTDYNDYALYYLCTA PTGGTPHDTYLIARRKPG-DNIPPQLESFTQGMEFKK--------C------
>G8JKD0	E---MVTVAECGGAQVNAMDNYHASDFHKEKWYVTHVTKVTEPTECRTVTATTKSDGKTF TVEHPFGEGGSQTLHCEAQAEAAKRLTFTCKVGDQVVDKTIFITVNTDYNDYALYYLCTA PTGGTPHDTYLIARRKAG-DNIPPQLESFTQGMDFKK--------C------
>G8JKC6	E---MVTVAECGGAQVNAMDNYHASDFHKEKWYVTHVTKVTEPTECRTVTATTKSDGKTF TVEHPFGEGGSQTLHCEAQAEAAKRLTFTCKVGDQVVDKTIFITVNTDYNDYALYYLCTA PTGGTPHDTYLIARRKPG-DNIPPQLESFTQGMEFKK--------C------
>G8JKG0	G------AECGGAQVNAMDNYHASDFHKEKWYVTHVTKVTEPTECRTVTATTKSDGKTF TVEHPFGEGGSQTLHCEAQAEAAKRLTFTCKVGDQVVDKTIFITVNTDYNDYALYYLCTA PTGGTPHDTYLIARRKPG-DNIPPQLESLTEGMDFKK--------C------
>G8JKC8	E---MVTVAECGGAQVNAMDNYRAPDYHKEKWYVTHVTKVTEPTECRTLTATTKSDGKTF TVEHPFGDGGSQTLHCEAQAEAAKRLTFTCKVGDQVVDKTIFITVNTDYNDYSLYYLCIA PTGGTPHDTYLIARRKPGDDNIPPQLESLTEGMDFKK--------C------
>G8JKD3	E---MVTVAECGGAQVNAMDNYRAPDYHKEKWYVTHVTKVTEPTECRTLTATTKSDGKTF TVEHPFGDGGSQTLHCEAQAEAAKRLTFTCKVGDQVVDKTIFITVDTDYNDYSLYYLCIA PTGGTPHDTYLIARRKPD-DNIPATLKELTSGKDFKK--------CSDLTK-
>G8JKF7	--------------KVNAMDNYHAPDFHKDKWYVTHVSKVTDPTECRTLTATNSADGKTF TVENSVGEEEGQKSTCVGHEEAANKLSFTCKVGNQVVAKSILITVATDYKDYALIYICST PTGRSPMNPYLIARRKPD-DNIPEALKELTKGKDFKK--------CSDLTK-
>G8JKF6	--------------KVNAMDNYHAPDFHKDKWYVTHVSKVTDPTECRTLTATNSADGKTF TVENSVGEEESQKSTCVGHEEAANKLSFTCKVGNQVVAKSILITVATDYKDYALIYICST PTGRSPMNPYLIARRKPD-DNIPEALKELTKGKDFKK--------CSDLTK-
>G8JKF8	--------------TVNAMDNYHAPDFHKDKWYVTHVSKVTDPTECRTLTATNSADGKTF TVENSVGEGESQKSTCVGHEEAANKLSFTCKVGNQVVAKSILITVATDYKDYALIYICST PTGRSPMNPYLIARRKPD-DNVPEALKELTKGKDFKK--------CSDLTK-
>G8JKG5	------TVAECGGEQVNAMDNYRAPDFHKDKWYVTHVSKVTDPTECRTLTATNSADGKTF TVENSVGEEESQKNTCVGHEEAANKLSFTCKVGNQVVAKSILITVATDYKDYALIYICST PTGRSPMNPYLIARRKPD-DNIPEALKELTKGKDFKK--------CSDLTK-

>G8JKD1	E---MITVAECGGEQVNAMDNYHAPDFHKDKWYVTHVTKVTEPTECRTLTATNSEDGKTF<br>TVENSVGEGESQKSTCVGHEEAANKLSFTCKVGNQVVAKSILITVATDYKDYALIYICST<br>PTGRSPMNTYLIARRKAG-DNIPPQLESFTQGMEFKK--------C------
>G8JKF9	--------------TVNAMDNYHAPDFHKDKWYVTHVTKVIEPTECRTLTATNSEDGKTF<br>TVENSVGEGESQKSTCVGHEEAANKLSFTCKVGNQVVAKSILITVATDYKDYALIYICST<br>PTGRSPMNTYLIARRKAG-DNIPPQLESFTQGMEFKK--------C------
>G8JKE6	E---MITVAECGGEQVNAMDNYHAPDFHKDKWYVTHVTKVTEPTECRTLTATNSEDGKTF<br>TVENSVGEGESQKSTCVGHEEAANKLSFTCKVGNQVVAKSILITVATDYKDYALIYICST<br>PTGGTPHDTYLIARRKPG-DNIPPQLESFTQGMEFKK--------C------
>G8JKG3	--------------QVNAMDNYHAPDFHKDKWYVTHVTKVTEPTECRTLTATNSADGKTF<br>TVENSVGEGESQKSTCVGHEEAANKLSFTCKVGNQVVAKSILITVATDYKDYALIYICST<br>PTGRSPMNTYLIARRKAG-DNIPPQLESFTQGMEFKKMLVKNKNECPKTFVV
>G8JKG4	--------------QVNAMDNYHAPDFHKDKWYVTHVTKVTEPTECRTLTATNSADGKTF<br>TVENSVGEGESQKSTCVGHEEAANKLTFTCKVGDQVVDKTIFITVNTDYNDYALYYLCTA<br>PTGGTPHDTYLIARRKAG-DNIPPQLESFTQGMEFKK--------C------
>G8JKG9	RAYSEDPFSKC-SKPKTVMDDYDATKWHSGKWYVTHVQKESTPTDCRTL--TTSQDGDVS<br>IVQHPY-ETGNGTLYCQGKKQEDNSLIFDCKSGEESMDKTIYIAVDTDYTDYALYYLCTS<br>PTKGDLYENYLVARRKEGQKDIPQQLQSSTSSLNLKQ--------CK-----
>G8JKD5	----EDPFSKC-SKPKTVMDDYDATKWHSGKWYVTHVQKESTPTDCRTL--TTSQDGDVS<br>IVQHPY-ETGNGTLYCQGKKQEDNSLIFGCKSGEESMDKTIYIAVDTDYTDYALYYLCTS<br>PTKGDLYENYLVARRKEGQKDIPQQLQSSTSSLNLKQ--------CK-----
>G8JKG8	-------------------DDYDATKWHSGTWYVTHVQKESTPTDCRTL--TTSQDGDVS<br>IVQHPY-ESGNGTLYCQGKKQEDNSLIFDCKSGDESMDKTIYIAVATDYNNYALYYLCTS<br>PTTGDLYENYLVARRKEGQKDIPQQLQSSTSSLNLKQ--------CK-----
>G8JKD4	-----DQFSAC-KKPTTVMDGYDATKWHTGTWYVTHVQKESTPTDCRTL--TTSQDGDVS<br>IVQHPY-ETGNGTLYCQGKKQEDNSLIFDCKSGDESMDKTIYIAVATDYNNYALYYLCTS<br>PTTGDLYENYLVARRQDGQKDIPKQLQSSTSSLNLKQ--------C------
	D. maxima
>G3CJQ3	------------------------------DFKATQFHSGTWYVTHVANETEPTDCRTLS<br>MSTTTSG-NSKTFVVQHPYGEGDKDKLHCTAQPETEKRLTFTCKNGGKVTDTTIFIAMVT<br>DYNDYALYYLCTTVKSGSNEGKVYDNYLVARRDGSKKDIPAALKTYTNNLGLKSC-

>G3CJQ4	TFIALTFIGILTYA-HGA-SECKTPTPVVPDFKATQFHSGTWYVTHVANETEPTDCRTLS<br>MSTTTSG-NSKTFVVQHPYGEGDKDKLHCTAQPETEKRLTFTCKSGGKVTDTTIFIAMVT<br>DYNDYALYYLCTTVKSGSNEGKVYDNYLVARRDGSKKDIPAALKTYTNNLGLKSC-
>G3CJP4	---------------------------------AGDFHSGTWYVTHVANETEPTDCRTLS<br>MSTTTSG-NSKTFVVQHPYGEGDKDKLHCTAQPETEKRLTFTCKSGGKVTDTTIFIAMVT<br>DYNDYALYYLCTTVKSGSNEGKVYDNYLVARRDGSKKDIPAALKTYTNNLGLKSC-
>G3CJR4	-----------------A-SECKTPTPVVQDFKATDFHSGTWYVTHVANETEPTDCRTLS<br>MSTTTSG-NSKTFVVQHPYGEGDKDKLHCTAQPETEKRLTFTCKSGGKVTDTTIFIAMVT<br>DYNDYALYYLCTTVKSGSNEGKVYDNYLVARRDGSKKDIPAALKTYTNNLGLKSC-
>G3CJQ2	TFIALTFIGILTYA-HGA-SECKTPTPVVQDFKATDFLSGTWYVTHVANQTDPTDCRTLS<br>MSTTGSG-NSKTFVVQHPYGEGDKDQLHCTAQPETEKRLTFTCKSGGKVTDTTIFIAMVT<br>DYNDYALYYLCTTVKSGSNEGKVYDNYLVARRDGSKKDIPAALKTYTNNLGLKSC-
>G3CJN4	---------------------------------ATDFHSGTWYVTHVANQTDPTDCRTLS<br>MSTTGSG-NSKTFVVQHPYGEGDKDQLHCTAKPETEKRLTFTCKSGGKVTDTTIFIAMVT<br>DYNDYALYYLCTTVESGSSKGQIYDNYLVARRDGSKKDIPAALKTYTNNLGLKSC-
>G3CJM4	----ITFIGILTYA-HGA-SECKTPTPVVQDFKATDFHSGTWYVTHVANQTDPTDCRTLS<br>MSTTGSG-NSKTFVVQHPYGEGDKDQLHCTAKPETEKRLTFTCKSGGKVTDTTIFIAMVT<br>DYNDYALYYLCTTVESGSSKGQIYDNYLVARRNGSKKDIPAALKTYTNNLGLKSC-
>G3CJP0	------------------------------DFKATDFHSGTWYVTHVANETEPTDCRTLS<br>MSTTTSG-NSKTFVVQHPYGEGDKDKLHCTAQPETEKRLTFTCKSGGKVTDTTIFIAMVT<br>DYNDYALYYLCTTVKSGSNEGKVYDNYVVARRSQT-KEIPEKLKSSTKDLDMKPC-
>G3CJQ5	-------------A-GGSISECKTPASVVDDFSATKFHGGTWYVTHVANQTDPTECRTLT<br>TSTTGSG-KDKTYVVQHPFGDGDKETLRCTAQPEQQKRLTFTCKNGETVTDTTIFIAMVT<br>DYNDYALYYLCTTVKSGSNEGKVYDNYLVARRDGSKKDIPAALKTYTNNLGLKSC-
>G3CJR5	-----------------SISECKTPASVVDDFSATKFHGGTWYVTHVANQTDPTECRTLT<br>TSTTGSG-KDKTYVVQHPFGDGDKETLRCTAQPEQQKRLTFTCKNGETVTDTTIFIAMET<br>DYNDYALYYLCTTVKAGDKNGQIYDNYVVARRSKT-NEIPDKLKSLTKDLNMKPC-
>G3CJS5	----------------GSISECKKPQP-MNGFSGTQFHGGVWYVTHVSNVTDPTECRTLT<br>TSKVG-----EKYIVEHPFESGD-GKLRCEATGEAEKRLTFTCKTGGTVTDSTIFIAMDT<br>DYNDYALYYLCTTVKAGDKMGEIYDNYVVARRSQT-KEIPEKLKSSTKDLDMKPC-
>G3CJN7	------------YAYAGSISECKKPQP-MNGFSGTQFRGGVWYATHVSNVTDPTECRTLT<br>TSKVG-----EKYIVEHPFESGD-GKLRCEATGEAEKRLTFTCKTGGTVTDSTIFIAMDT<br>DYNDYALYYLCTTVKAGDKMGEIYDNYVVARRSQT-KEIPEKLKSSTKDLDMKPC-

>G3CJM3	--------GILTYVYAGSISECKTPKP-MDDFSGTKFHGGIWHVTHVANVTDPTECRTLT<br>TSKVG-----EKYIVEHPFESSD-GTLRCEATGEAEQKLTFTCKTGSTVTDTTIFIAMET<br>DYEDYALYYLCTTVKSS---GDMYDNYVVARRSPS-KEIPGKLKSLTKDLGLEPCS
>G3CJR7	---------------GSISECKQPTPVVDGFKATEFHTGTWYVTHVANKTEPTDCRTLS<br>TSTRNPGTNDKTYIVEHPYEVGNEKKLHCEAKPEAQKRLTFACKTDGKDTDSTIFIAMAT<br>DYKDYALYYLCTTVLSGSDKGQKYDNFLVARRSPS-NEIPGKLQDLTKNLNLQRCS
	T. brasiliensis
>Q0MTD4	YAYAATTGISQCQTVTPMEGFSATSFFTGTWYVTHVQKNTSATVCQTFTTKDENRTLVVE<br>YSF-NDGQQKNVRCEG-QRGEEKKVAFNCKVNEAPMFDVDFIVLATDYDDYALFYRCITF<br>TSSGDKNDNYLVLRRSSGDQEIPASLRDLTSSLNLQSCQNLRASVL
>Q0MTC6	----ATTGISECQNVTPMERFSATQFFTGTWYVTHVQKNTSATVCQTFTTKDENRTLVVE<br>YSF-NDGQENNVRCEGQQRRRKKKLHFKCKVNDVPKFDADFIILDTDYNDYALFYRCITF<br>TSSGSKNDNYLVLRRSSGDQDIPTSLASLTSKLGLLAC--------
>Q0MTD9	----ATTGISECQNVTPMERFSATQFFTGTWYVTHVQKNTSATVCQTFTTKDENRTLVVE<br>YSF-NDGQENNVRCEG-QRGEEKKVAFKCKVNDVPKFDADFIILDTDYNDYALFYRCITF<br>TSSGSKNDNYLVLRRSSGDQDIPTSLASLTSKLGLLACKDIRPPVA
>Q0MTE9	----ATTGISECQNVTPMERFSATQFFTGTWYVTHVQKNTSATVCQTFTTKDENRTLVVE<br>YSF-NDGQENSVRCEG-QRGEEKKVAFKCKVNDVPKFDADFIILDTDYNDYALFYRCITF<br>TSSGSKNDNYLVLRRSSGDQDIPTSLASLTSKLGLLACKDIRPPVA
>Q0MTC9	-----------CQTVTPMDGFTATRFFTGTWYVTHVQKNTSATVCQTFTTKDENRTLVVE<br>YSFNNNGQENKVRCEG-QRGVEKKVAFNCKVNGAHTFDADFIVLDTDYTDYALFYRCVTF<br>T-SGSKDDNHLVLRRSPGIQQIPESLTSLTSGLGLMSCE-------

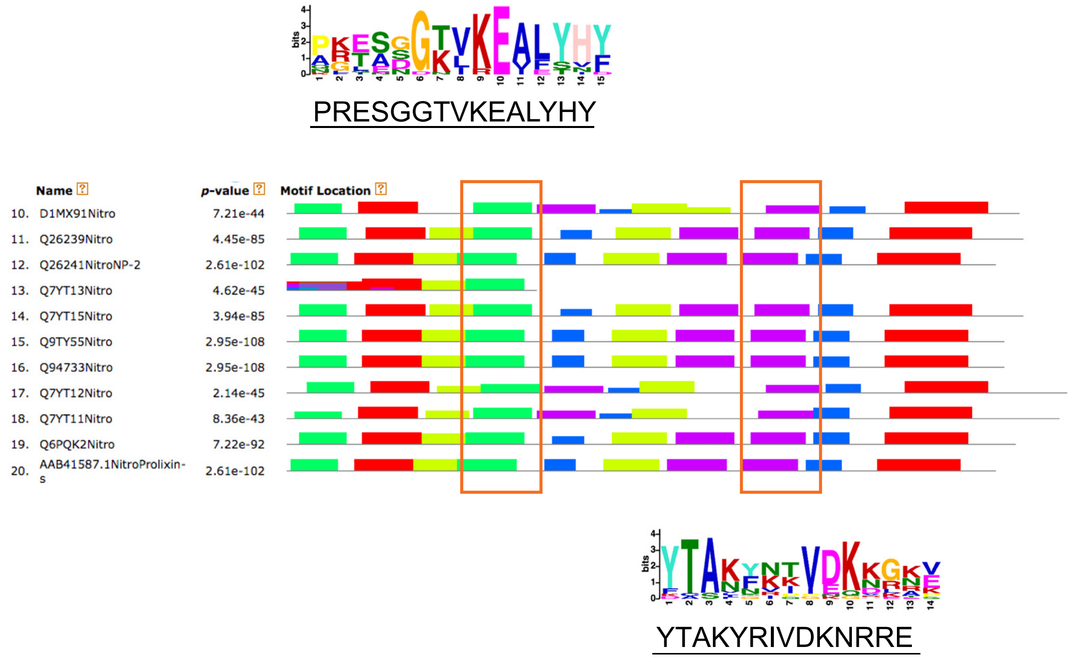

Figure S1. Representative amino acid sequence alignment of nitrophorins and amine binding proteins. The sequence alignments were performed with the MEME software, and they show the sequence motif detected and their position within the protein sequence (The amino acid sequences in FASTA format are part of the supplementary data, see Table S3).

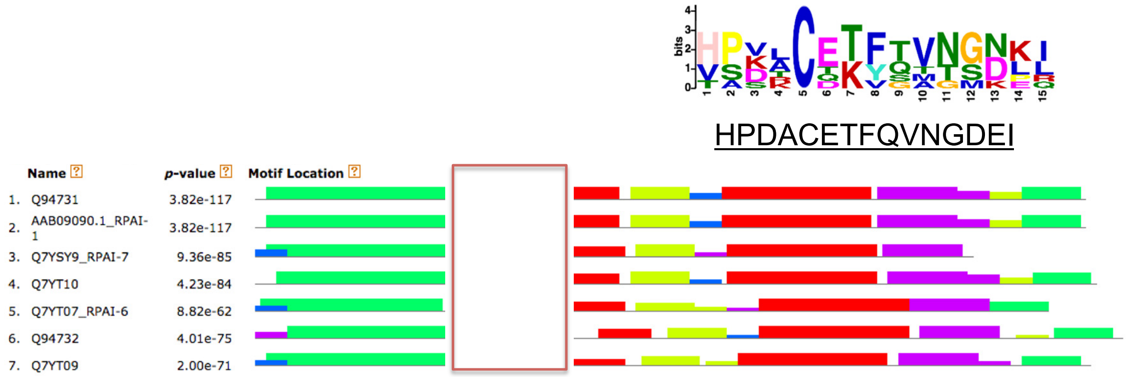

Figure S2. Representative amino acid sequence alignment of *Rhodnius prolixus* aggregation inhibitors (RPAI). The sequence alignments were performed with the MEME software, and they show the sequence motif detected and their position within the protein sequence (The amino acid sequences in FASTA format are part of the supplementary data, see Table S3).

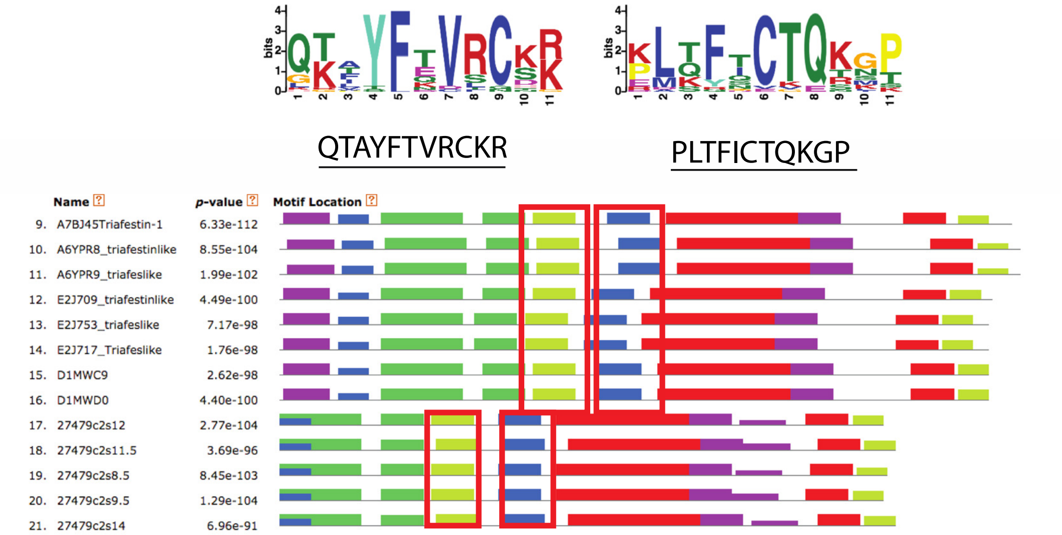

Figure S3. Representative amino acid sequence alignment of triafestins. The sequence alignments were performed with the MEME software, and they show the sequence motif detected and their position within the protein sequence (The amino acid sequences in FASTA format are part of the supplementary data, see Table S3).

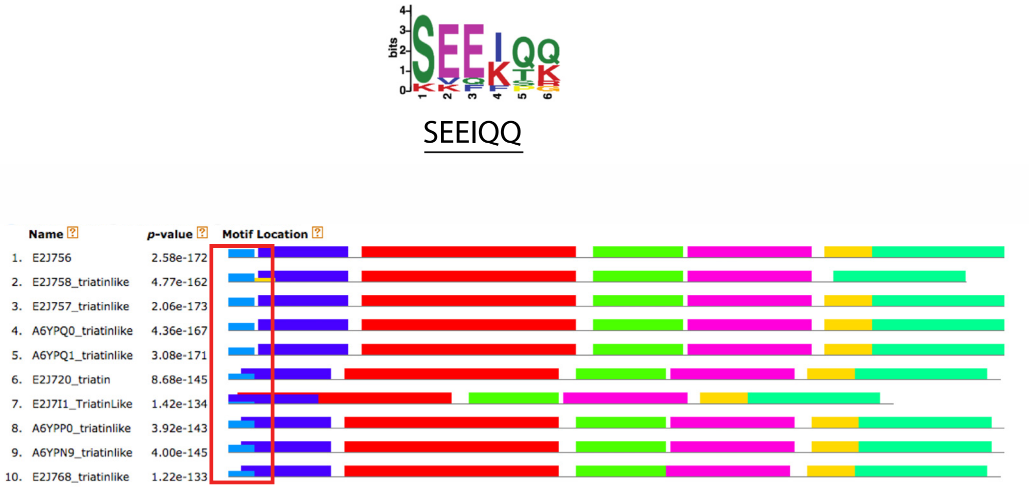

Figure S4. Representative amino acid sequence alignment of triatins. The sequence alignments were performed with the MEME software, and they show the sequence motif detected and their position within the protein sequence (The amino acid sequences in FASTA format are part of the supplementary data, see Table S3).

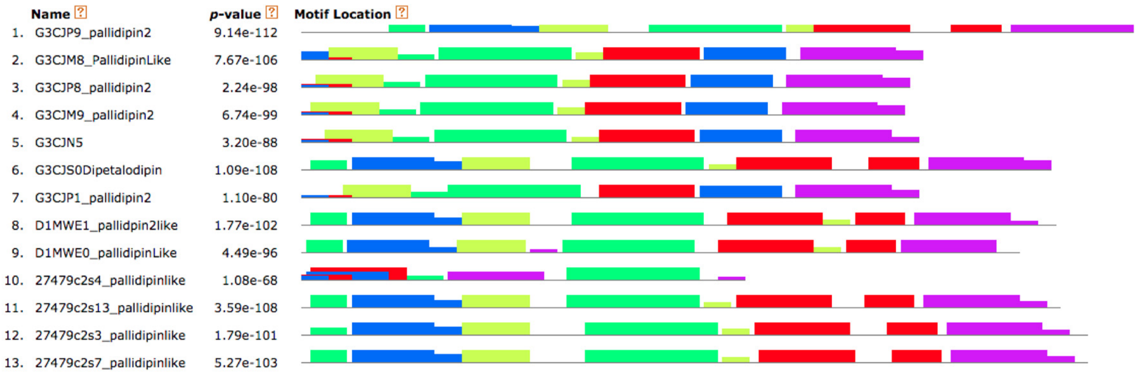

Figure S5. Representative amino acid sequence alignment of pallidipins and dipetalopidins. The sequence alignments were performed with the MEME software. However, an exclusively sequence motif for this clade was not found (The amino acid sequences in FASTA format are part of the supplementary data, see Table S3).

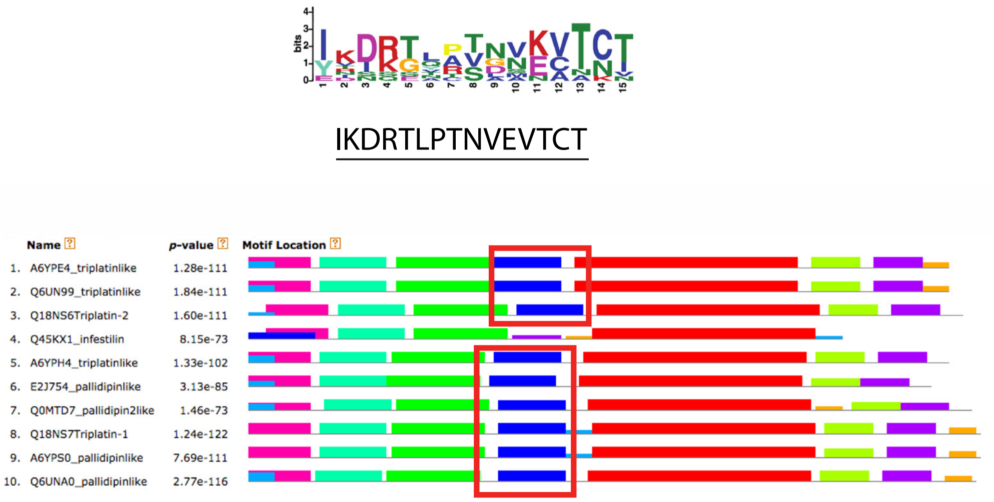

Figure S6. Representative amino acid sequence alignment of triplatins and infestilin. The sequence alignments were performed with the MEME software, and they show the sequence motif detected and their position within the protein sequence (The amino acid sequences in FASTA format are part of the supplementary data, see Table S3).

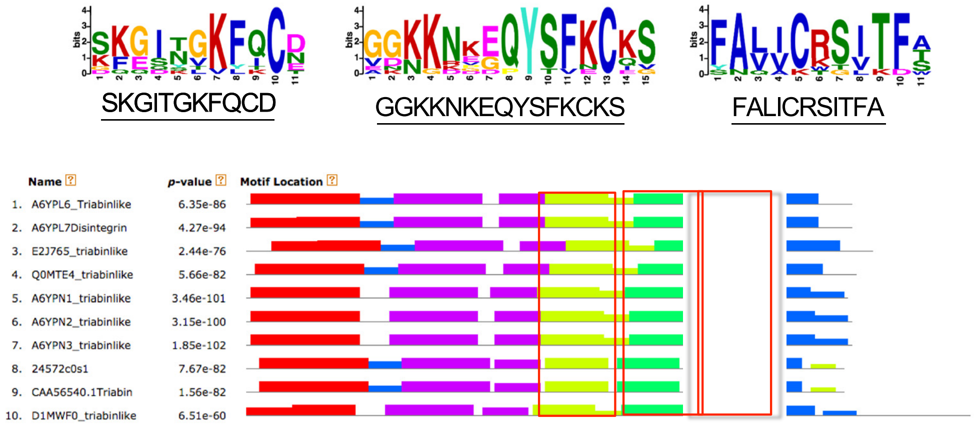

Figure S7. Representative amino acid sequence alignment of triabins and dimiconins. The sequence alignments were performed with the MEME software, and they show the sequence motif detected and their position within the protein sequence (The amino acid sequences in FASTA format are part of the supplementary data, see Table S3).

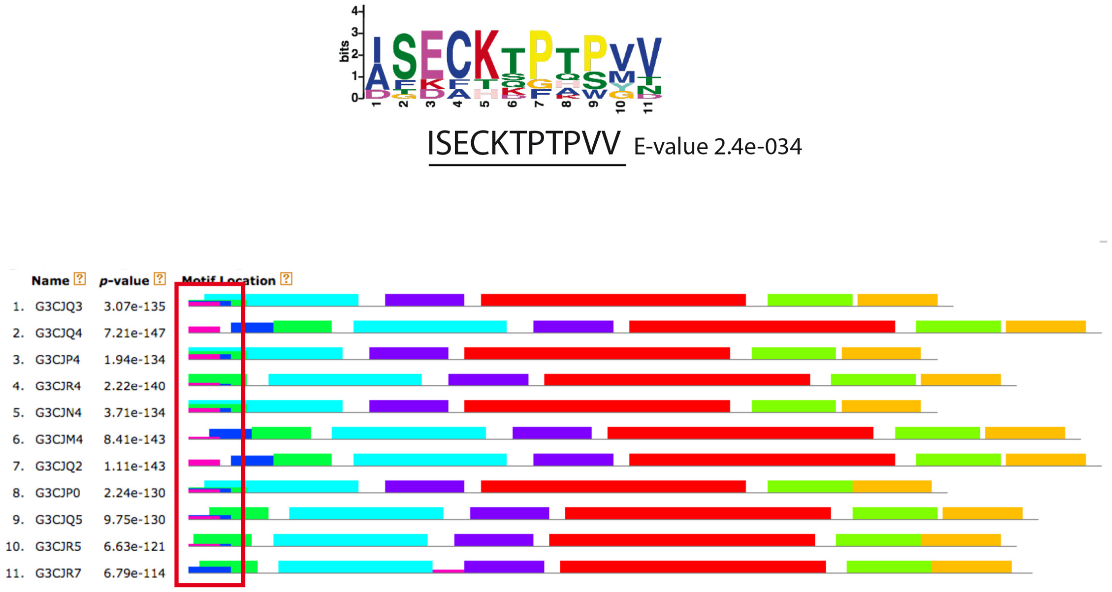

Figure S8. Representative amino acid sequence alignment of triabins from *D. maxima*. The sequence alignments were performed with the MEME software, and they show the sequence motif detected and their position within the protein sequence (The amino acid sequences in FASTA format are part of the supplementary data, see Table S3).

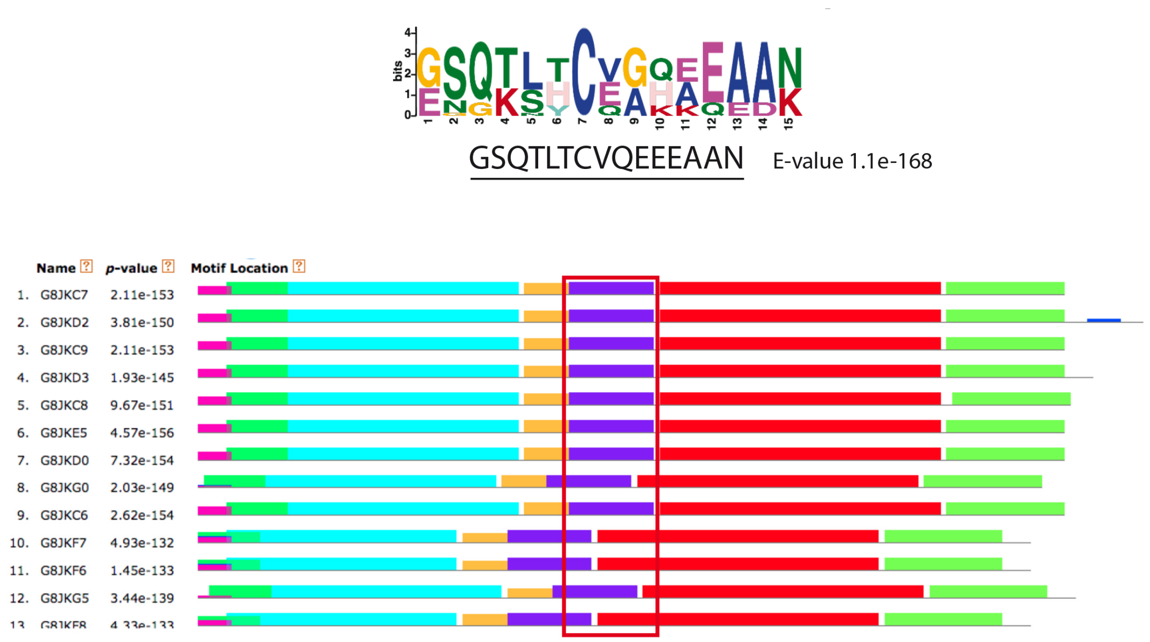

Figure S9. Representative amino acid sequence alignment of triabins from *T. rubida*. The sequence alignments were performed with the MEME software, and they show the sequence motif detected and their position within the protein sequence (The amino acid sequences in FASTA format are part of the supplementary data, see Table S3).

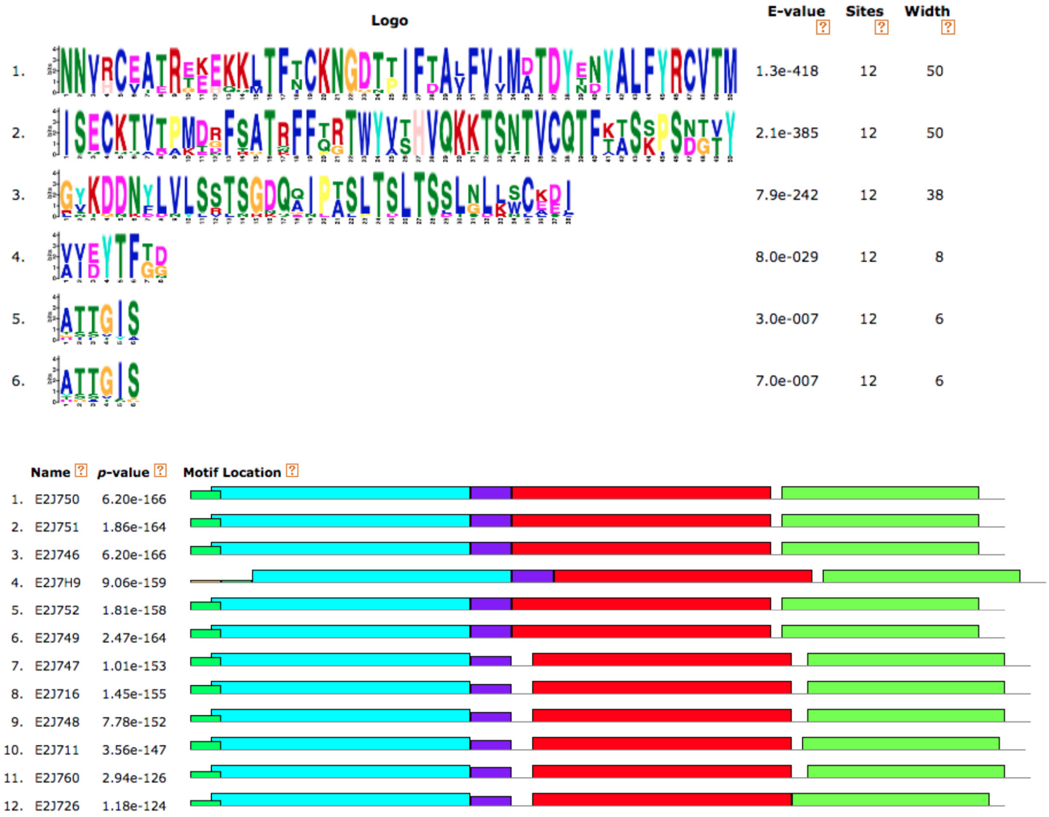

Figure S10. Representative amino acid sequence alignment of triabins from *T. matogrossensis*. The sequence alignments were performed with the MEME software. There was not a specific sequence motif, but the graphical representation of the sequence conservation (logo) is shown (The amino acid sequences in FASTA format are part of the supplementary data, see Table S3).
